# Experience and impact of stigma in people with chronic hepatitis B: a qualitative study in Asia, Europe, and the United States

**DOI:** 10.1186/s12889-023-17263-6

**Published:** 2024-02-26

**Authors:** Mondher Toumi, Jack Wallace, Chari Cohen, Chris Marshall, Helen Kitchen, Jake Macey, Hannah Pegram, Ashley F. Slagle, Robert G. Gish, Qin Ning, Hiroshi Yatsuhashi, Markus Cornberg, Maurizia Brunetto, Florian van Bömmel, Qing Xie, Dee Lee, Noriyuki Habuka, Urbano Sbarigia, Maria Beumont-Mauviel, Angelina Villasis Keever, Yasushi Takahashi, Yiwei Lu, Ao Liu, Qiaoqiao Chen, Tetsuro Ito, Olaf Radunz, Anna Puggina, Gudrun Hilgard, Eric K.H. Chan, Su Wang

**Affiliations:** 1 Aix-Marseille University, Jardin du Pharo, 58 bd Charles Livon, Marseille, 13284 Cedex 07 France; 2 Burnet Institute, 85 Commercial Rd, Melbourne, VIC 3004 Australia; 3 Hepatitis B Foundation, 3805 Old Easton Rd, Doylestown, PA 18902 USA; 4 Clarivate (formerly DRG Abacus), 70 St Mary Axe, London, EC3A 8BE UK; 5 Aspen Consulting, LLC, 625 S Lincoln Ave #101, Steamboat Springs, CO 80487 USA; 6 Tongji Hospital, Tongji Medical College, Huazhong University of Science and Technology, 1095 Jie Fang Avenue, Hankou, Wuhan, 430030 China; 7 National Hospital Organization (NHO) Nagasaki Medical Center, 2-1001-1, Kubara, Omura, Nagasaki 856-8562 Japan; 8 Nagasaki University Graduate School of Biomedical Sciences, 1-7-1 Sakamoto, Nagasaki City, 852-8520 Japan; 9 Hannover Medical School, Carl-Neuberg-Str. 1, 30625 Hannover, Germany; 10 University Hospital of Pisa, Lungarno Pacinotti 43, Pisa, 56126 Italy; 11 Division of Hepatology, Department of Medicine II, Leipzig University Medical Center, Liebigstrasse 20, 04103 Leipzig, Germany; 12 Shanghai Jiao Tong University School of Medicine, Ruijin Hospital, 227 South Chongqing Road, Shanghai, 20025 China; 13 Inno Community Development Organisation, Room 208, Dengzheng Business Center, #57, Dengzhengnan Rd, Yuexiu District, Guangzhou, Guangdong China; 14 Janssen Pharmaceutical K.K, 3-5-2 Nishi-kanda, Chiyoda-ku, Tokyo, 101-0065 Japan; 15 Janssen Pharmaceutica NV, Turnhoutseweg 30, Beerse, B-2340 Belgium; 16 Janssen Research & Development, LLC, 1125 Trenton Harbourton Rd, Titusville, NJ 08560 USA; 17 Janssen Research & Development, 1400 McKeon Road, Spring House, PA 19002 USA; 18 Janssen China, 14F, Tower 3, China Central Place, No.77, Jian Guo Road, Chaoyang District, Beijing, 100025 China; 19 Janssen Health Economics & Market Access (EMEA), 50-100 Holmers Farm Way, High Wycombe, Buckinghamshire, HP12 4EG UK; 20 Janssen Germany, Johnson-u.-Johnson-Platz 1, 41470 Neuss, Nordrhein-Westfalen Germany; 21 Janssen Italy, Via Michelangelo Buonarroti, 23, Cologno Monzese, 20093 Italy; 22 Janssen Global Services, LLC, 1000 US 202, Raritan, NJ 08869 USA; 23 Cooperman Barnabas Medical Center, 222 Columbia Turnpike, Florham Park, NJ 07932 USA; 24 Department of Public Health, Aix-Marseille University, 27 Boulevard Jean Moulin, Marseille, 13385 France

**Keywords:** Hepatitis B virus, Chronic Hepatitis B, Functional cure, Qualitative research, Self-stigma, Social impact

## Abstract

**Background:**

People with chronic hepatitis B (CHB) commonly experience social and self-stigma. This study sought to understand the impacts of CHB-related stigma and a functional cure on stigma.

**Methods:**

Adults with CHB with a wide range of age and education were recruited from 5 countries and participated in 90-minute qualitative, semi-structured interviews to explore concepts related to CHB-associated stigma and its impact. Participants answered open-ended concept-elicitation questions regarding their experience of social and self-stigma, and the potential impact of reduced CHB-related stigma.

**Results:**

Sixty-three participants aged 25 to 71 years (15 from the United States and 12 each from China, Germany, Italy, and Japan) reported emotional, lifestyle, and social impacts of living with CHB, including prejudice, marginalization, and negative relationship and work experiences. Self-stigma led to low self-esteem, concealment of CHB status, and social withdrawal. Most participants stated a functional cure for hepatitis B would reduce self-stigma.

**Conclusions:**

CHB-related social and self-stigma are widely prevalent and affect many aspects of life. A functional cure for hepatitis B may reduce social and self-stigma and substantially improve the health-related quality of life of people with CHB. Incorporating stigma into guidelines along with infectivity considerations may broaden the patient groups who should receive treatment.

**Supplementary Information:**

The online version contains supplementary material available at 10.1186/s12889-023-17263-6.

## Background

Hepatitis B virus (HBV) is a major global health concern; an estimated 296 million people worldwide were living with chronic hepatitis B (CHB) in 2019 [1, 2]. Stigma and discrimination are often experienced by people living with CHB, particularly in some Asian and African countries where HBV prevalence is high and several Asian immigrant communities in North America [3–7] and Australia [7, 8].

HBV-associated social stigma describes situations where people stigmatize those with hepatitis B [9–12], and can present as discrimination, judgment, marginalization, or denial of treatment [4–6, 13–16].

Self-stigma refers to when people with CHB internalize negative beliefs, which negatively impacts their emotions and scope of activities [9, 17–19]. Experiencing discrimination (i.e. social stigma) is associated with negative feelings, self-blame, and isolation (i.e. self-stigma) and lowers health-related quality of life (HRQoL) [12, 13, 15, 20]. The psychosocial effects of self-stigma, such as fear of disease progression or of transmitting the infection and potential consequent shame, depression, and anxiety, all substantially impact people living with CHB [12, 15–17, 21, 22].

CHB-related self-stigma hampers diagnosis and effective treatment by reducing willingness to present for diagnostic and clinical monitoring [6, 12, 22–24] and treatment initiation and adherence. This can increase likelihood of transmission [15] and poses a barrier for viral hepatitis elimination [25]. Hence, understanding, reduction, and, ultimately, elimination of CHB-associated stigma are critical [26]. Stigma is not currently modeled into decision-making for screening and treatment guidelines.

In moving to a functional cure treatment program for HBV [27] and an era emphasizing patient focus in drug development [28], it is important to have a thorough understanding of social and self-stigma and their impact on the lives of people with HBV and the management and treatment of CHB. This qualitative study was conducted to comprehensively understand experiences of social and self-stigma among people with CHB in countries with varying HBV prevalence in Asia, Europe, and North America, and identify the impact of a potential functional cure for HBV on self-stigma.

## Methods

Qualitative semi-structured interviews exploring CHB-associated stigma and its impact on daily life were used. Adults aged ≥ 18 years with clinically confirmed CHB who were either treatment naïve or treated were recruited from China, Germany, Italy, Japan, and the United States. Additionally, in China and Japan, adults aged ≥ 18 years with prescription, doctor’s note, or participant-provided photographs of medication were also recruited in the study. Exclusion criteria included having other clinical conditions associated with self-stigma (e.g. chronic hepatitis C, HIV), and psychiatric or other conditions that might impair ability to participate in the study.

Participants were recruited by a recruitment agency using existing patient associations and social media and were reimbursed for time and travel. Purposive sampling aimed to recruit an ethnically diverse and representative sample including people of different ages, employment statuses, and educational backgrounds.

Each individual participated in a 90-minute, semi-structured interview (face-to-face or telephone/web-assisted) in the local language, conducted by trained qualitative interviewers. The objectives were to understand and document experience of CHB-associated social and self-stigma, using a semi-structed interview guide that comprised open-ended concept-elicitation questions and probes regarding (i) feelings, experiences, and events of stigma and self-stigma; (ii) experiences of social stigma that influence self-stigma; (iii) the most important and/or bothersome aspects of self-stigma; (iv) impact on HRQoL and CHB management; and (v) potential impacts on daily life of removing or reducing social and self-stigma.

Conceptual saturation analysis was performed based on International Society for Pharmacoeconomics and Outcomes Research (ISPOR) Task Force Best Practice Guidelines for Establishing Content Validity [29]. For concept analysis, interview transcripts were divided into 3 small sets for each country (*n* = 4–5 in each set) in the sequential order in which they were performed; spontaneously elicited self-stigma concepts were compared. Concepts arising in Set 1 and Set 2 (not Set 3) would indicate saturation had been achieved. Analyses based on thematic methods were conducted using ATLAS.ti software (ATLAS.ti Scientific Software Development GmbH, Berlin, Germany) [30, 31].

The study protocol was reviewed and approved by relevant institutional ethics review boards, and participants completed an informed consent form before being interviewed.

## Results

### Participants

Sixty-three people with CHB (15 from the United States and 12 each from China, Germany, Italy, and Japan) participated in interviews between February 2019 and March 2020. The mean age was 49 (range 25–71) years, and 62% were female (Table 1). In all countries, participants had a wide range of educational backgrounds, employment statuses, treatment modalities, and treatment durations (Table 1). In the United States, more participants reported Asian ethnicity (36%) than in European countries (8% in Germany and 0% in Italy).

**Table 1:** Demographic and clinical characteristics of participants

	United States (*n* = 15^a^ )	Germany (*n* = 12^b^)	Italy (*n* = 12)	China (*n* = 12)	Japan (*n* = 12)	Total (*N* = 63)
**Sex, n (%)**						
Female	8 (53)	7 (58)	8 (67)	7 (58)	9 (75)	39 (62)
Male	7 (47)	5 (42)	4 (33)	5 (42)	3 (25)	24 (38)
**Mean age, years**^**c**^	36	48.8	53.4	38	58.1	48.8
**Median age (range), years**^**c**^	49.5 (36–58)	52 (31–65)	58.5 (29–71)	38 (25–57)	59.5 (44–70)	50.0 (25–71)
**Ethnicity, n (%)**						
Asian	5 (36)	1 (8)	0	12 (100)	12 (100)	30 (48)
Black/African American	1 (7)	0	0	0	0	1 (2)
Caucasian/White	6 (43)	10 (83)	12 (100)	0	0	28 (44)
Hispanic	2 (14)	0	0	0	0	2 (3)
Other	0	1 (8)	0	0	0	1 (2)
**Living status, n (%)**						
Living with partner/spouse	4 (29)	3 (25)	2 (17)	9 (75)	4 (33)	22 (35)
Living with partner and children	5 (36)	1 (8)	5 (42)	0	5 (42)	16 (25)
Living with children	1 (7)	1 (8)	2 (17)	0	0	4 (6)
Living with family	1 (7)	0	1 (8)	1 (8)	1 (8)	4 (6)
Living alone	3 (21)	7 (58)	2 (17)	1 (8)	1 (8)	14 (22)
Living with parents	0	0	0	1 (8)	0	1 (2)
Other	0	0	0	0	1 (8)	1 (2)
**Highest education level, n (%)**						
Junior high school	0	0	0	0	2 (17)	2 (3)
High school	1 (7)	0	1 (8)	4 (33)	1 (8)	7 (11)
Technical college	0	0	0	0	4 (33)	4 (6)
Apprenticeship	0	2 (17)	0	0	0	2 (3)
Vocational training	0	5 (42)	0	8 (67)^d^	0	13 (21)
College or associate’s degree	6 (40)	1 (8)	7 (58)		0	22 (35)
University	0	4 (33)	1 (8)		5 (42)	18 (29)
Bachelor’s degree	2 (13)	0	1 (8)		0	11 (17)
Graduate or postgraduate degree	6 (40)	0	2 (17)	0	0	8 (13)
**Employment status, n (%)**^**e**^						
Employed full-time	12 (80)	5 (45)	2 (17)	9 (75)	6 (50)	34 (55)
Employed part-time	2 (13)	3 (27)	0	0	3 (25)	8 (13)
Unemployed	1 (7)	1 (9)	2 (17)	3 (25)	1 (8)	8 (13)
Retired	0	1 (9)	3 (25)	0	2 (17)	6 (10)
Other	0	1 (9)	4 (33)	0	0	5 (8)
**Mean time since diagnosis, years**^**f**^	10.7	10.5	11.7	10.6	19.7	12.5
**Genotype, n (%)**						
Genotype A	4 (27)	10 (83)	0	0	0	14 (22)
Genotype B	2 (13)	0	0	0	1 (8)	3 (5)
Genotype C	1 (7)	1 (8)	0	0	3 (25)	5 (8)
Genotype D	1 (7)	1 (8)	1 (8)	0	0	3 (5)
Unknown/NR	7 (47)	0	11 (92)	12 (100)	8 (67)	38 (60)
**Treatment naïve, n (%)**	2 (13)	4 (33)	3 (25)	0	1 (8)	10 (16)
**Current treatment, n (%)**						
NRTI	6 (40)	8 (67)	4 (33)	8 (67)	4 (33)	30 (48)
Tenofovir	2 (13)	6 (50)	NR	2 (17)	NR	10 (16)
Entecavir	4 (27)	2 (17)	NR	6 (50)	NR	12 (19)
Azathioprine	0	0	2 (17)	0	0	2 (3)
Unknown	0	0	0	0	2 (17)	2 (3)
Prednisone	0	0	1 (8)	0	0	1 (2)
Ursodiol	0	0	2 (17)	0	0	2 (3)
Furosemide	0	0	1 (8)	0	0	1 (2)
Interferon	2 (13)	1 (8)	0	3 (25)	2 (17)	8 (13)
Lamivudine	1 (7)	0	2 (17)	1 (8)	1 (8)	5 (8)
Tenofovir alafenamide	1 (7)	0	0	0	0	1 (2)
Adefovir dipivoxil	0	0	0	2 (17)	0	2 (3)
Thymopolypeptides	0	0	0	1 (8)	0	1 (2)
Traditional Chinese medicine	N/A	N/A	N/A	6 (50)	1 (8)	7 (11)
Unknown	2 (13)	0	0	1 (8)	1 (8)	4 (6)
Not currently on treatment	2 (13)	0	0	0	5 (42)	7 (11)
**Time since treatment initiation, n (%)**						
3–24 months	7 (47)	6 (50)	7 (58)	5 (42)	1 (8)	26 (41)
> 24 months	3 (20)	2 (17)	2 (17)	8 (67)	5 (42)	20 (32)
Unknown	1 (7)	0	0	0	0	1 (2)
**Comorbidities, n (%)**						
None	8 (53)	6 (50)	5 (42)	8 (67)	7 (58)	34 (54)
Depression	3 (20)^g^	0^ h^	0^ h^	0	1 (8)	4 (6)
Anxiety	2 (13)	0	1 (8)	0	0	3 (5)
High blood pressure	1 (7)	4 (33)	2 (17)	0	1 (8)	8 (13)
Heart disease	0	3 (25)	1 (8)	0	0	4 (6)
Congestive heart failure	0	2 (17)	1 (8)	0	0	3 (5)
Diabetes	0	2 (17)	4 (33)	0	2 (17)	8 (13)
Stroke	0	0	1 (8)	0	0	1 (2)
Asthma/respiratory disease	0	1 (8)	4 (33)	0	0	5 (8)
COPD	0	1 (8)	3 (25)	0	0	4 (6)
Thyroid disease	0	0	6 (50)	1 (8)	0	7 (11)
Arthritis/osteoarthritis	0	0	3 (25)	0	1 (8)	4 (6)
Cancer	0	0	1 (8)	0	2 (17)	3 (5)
Other	1 (7)	2 (17)	0	3 (25)	2 (17)	8 (13)

*COPD* Chronic obstructive pulmonary disease, *N/A* Not applicable in this jurisdiction, *NR* Not reported, *NRTI* Nucleoside analog reverse transcriptase inhibitor

^a^Age, ethnicity, and living status demographics for 14 US participants only; 1 had missing data

^b^Employment status data for 11 German participants only; 1 had missing data

^c^Total mean and median (range) age based on 62 participants

^d^University (bachelor’s degree) or vocational college

^e^Total employment n (%) based on 62 participants

^f^Total mean time since diagnosis based on 60 participants

^g^Participants’ depression under control and asymptomatic at the time of the interview

^h^*n*=4 reported multiple comorbidities

### Impact of living with CHB

Participants were asked to describe their experience of living with CHB. Five self-stigma concepts emerged: secrecy/concealment, withdrawal/social isolation, devaluation/inferiority/worthlessness, marginalization/alienation, and shame/guilt. Saturation was fully achieved (i.e. additional interviews would not provide new objective-relevant information [32]) in China, Germany, Italy, and the United States, with all 5 concepts spontaneously arising in the first or second set of interviews. In Japan, saturation was achieved for 3 of the 5 self-stigma concepts with devaluation/inferiority/worthlessness and marginalization/alienation not mentioned by any participants in Japan, likely due to participants not having disclosed their CHB status to others.

The concepts that emerged showed living with CHB has emotional, lifestyle, and social impacts (Table 2 and Additional File 1: Supplementary Table 1). Emotional impacts, such as anxiety/fear and embarrassment, were reported in all countries. Low self-esteem, secrecy/concealment, sadness, depression, difficulty accepting or being overwhelmed by their diagnosis, and feelings of anger, betrayal, denial, and not feeling “normal” were also reported.

**Table 2:** Concepts that emerged when participants were asked about their experience of living with CHB

Concept	Selected supporting quotes
**Emotional impacts**	
**Anxiety/fear** Reasons for feeling fearful included: • Transmission to family and friends • Disease progression, including liver function and cirrhosis • Treatment efficacy • Impact on work • Impact on relationships/fear of rejection	• *“The biggest concern is its transmission, as the disease is contagious.” (CN-09)* • *“Socially, I do think about being very cautious not to possibly spread this to my kids or anything when I’m using utensils around the house…” (US-14)* • *“I worry a lot about my liver. That it gets damaged…I’m quite young and I worry how many years I can live with it…I’m afraid of dying early.” (DE-09)* • *“I’m also worried about my condition getting worse. Because this disease is incurable currently, I just hope it isn’t getting worse.” (CN-10)* • *“I am afraid for my permanent job, how bad the illness is, whether I will get well again, whether the pills will help.” (DE-02)* • *“It’s like if…I had a time bomb in my pocket. I know I have it, but it’s unpredictable, so I do not know what could happen to me in the future or how it could affect my family, my children, my marriage.” (IT-11)* • *“ …some companies now do not require employees to take medical examination for hepatitis B, these companies may still require them to do so privately. If they are found to have hepatitis B, they may be fired…” I: And are you worried? “Of course.” (CN-10)* • *“Yes, definitely, especially in relation to my love and sexual life. Beyond precautions, because I don’t want to get pregnant or similar, I almost avoid being touched because I’m scared to be contagious, so I find it difficult, very complicated.” (IT-04)* • *“My approach and self-confidence when it comes to forming relationships, may that be romantic, or friendship, or business related…I don’t know if it’s subconscious at this point because I’ve done a lot of work over the years to try to get peace with my condition. But sometimes it does show up in weird ways with my behavior where I’ll sort of sabotage myself before getting to a point where I have to take a risk or something like that just for fear of rejection, mostly I think.” (US-12)*
**Shame/embarrassment about disclosing condition** • Includes shame related to inability to donate blood	• *“At the beginning I felt ashamed of telling people because I don’t really know when I got infected and how. And then I keep wondering about it. And people are asking.” (DE-09)* • *“For example, sometimes we see blood donation vehicles in the neighborhood and my friend asked when was the last time I donated blood? I did not know what to say.” (CN-02)*
**Low self-esteem**	• *“My self-esteem I think is a little bit down.” (US-13)*
**Need for secrecy/concealment**	• *“I never talk about my symptoms with my friends. So when I’m tired I blame other things for it.” I: So they don’t know about your illness? “No, definitely not.” (DE-11)* • *“Well, no one knows about it, only my office colleagues know about it. Only my family knows, but no one else. My partner doesn’t know. And that is it. It has always been something I didn’t want to share or talk about. You are very careful in certain situation but you don’t tell it. You can’t tell your friends, unless it is a real and close friend. You don’t talk about this topic, never.” (IT-01)* • *“In terms of social life, because basically I won’t share or discuss my disease with others, it’s a private thing. If you don’t tell, nobody knows that you have hepatitis B.” (CN-01)* • *“…it’s something that I’m not very comfortable with sharing with others.” (US-14)*
**Feeling overwhelmed, difficulty accepting diagnosis, sadness/depression, anger, betrayal, denial, not feeling human/normal**	• *“I was lost when I got diagnosed. I was overwhelmed.” (CN-03)* • *“Sometimes it can be such an emotional burden. I don’t consider myself a normal person anymore.” (CN-02)* • *“[The] month that it happened and I was a very upset, disturbed, and bothered person. I was irritable. I was angry.” (US-11)* • *“I felt betrayed. I felt abused by this guy that I had dated.” (US-11)* • *“I think there was a bit of denial of the beginning.” (US-15)* • *“It’s affected me emotionally by making me not feel human, making me not feel like I can do everything I want to do. It makes me feel like a lost soul. It makes me feel like just like I got this burden on me that I just can’t get rid of, and no matter what I do, I can’t shake it. It makes me feel kind of useless at times.” (US-10)*
**Lifestyle limitations**	
**Reduced/no exercise**	• *“Well, my lifestyle has changed, for sure, since I feel tired more frequently, I had to give up on a lot of sports.” (IT-12)* • *“I can’t do any heavy house chores.” (CN-06)*
**Dietary limitations and reduced/no alcohol intake**	• *“…The impact it has on living habits is that I need to pay more attention to my diet now.” (CN-04)* • *“When I went out with friends and everyone was drinking alcohol and I was not allowed to. And I felt as an outsider.” (DE-05)* • *“I have to avoid alcohol. I don’t mean to get drunk, but a glass of wine would be nice at dinner. Fatty foods as well. I was told off by all the specialists.” (IT-07)* • *“I never really drank much alcohol and now I won’t drink any because I always worry about the liver cirrhosis.” (US-03)*
**Increased hospital visits, frequent treatment, different attitudes from medical staff**	• *“I have to go for blood tests more periodically than the average person.” (US-03)* • *“The impact on life is that I have to take medicines on time every day.” (CN-01)* • *“The medications must be taken on an empty stomach or two hours after mealtime. Therefore, I cannot have a nightlife. I like to take my medications at nine o’clock and finish my meal at seven o’clock. It affects my social life.” (CN-07)* • *“Yes, I always state it [hepatitis B status]; I had an appointment in the morning and once they saw on the form that I have hepatitis B, I was taken as the last patient.” (DE-07)*
**Unable to share utensils**	• *“For example, if I have drunk from a glass or a bottle, I make sure that nobody else drinks from it.” (DE-01)* • *“I try not to eat the hot pot communally and share their dishes with others. I usually eat on individual dishes and don’t share plates and utensils.” (JP-12)* • *“I would use independent tableware, and my dishes were also placed on a separate plate. I use a toilet by myself. My clothes or towels, including my cups, toothbrush, are all kept separately. I wanted to do it myself because there is a child at home. I am very worried that it will be transmitted to my child.” (CN-11)* • *“Some of my relatives, middle-aged and elderly women, are generally mean-minded. When I went to their house for dinner, they will prepare a set of cutleries for me, and then said that they are prepared for me. After I used them up, they throw away those cutleries.” (CN-09)*
**Impact on work** • Taking time off work • Increased fatigue impacts performance • Some jobs not/no longer possible	• *“I get tired more easily. Fatigue is always there, so it has influenced my performance.” (IT-01)* • *“…right after my diagnosis. I did not go to work for half a year. Treatments took some time. Also because my numbers were too high so I couldn’t go back to work.” (CN-06)* • *“I have to see a doctor every other month or once every three months. I have to have lab tests all the time. I have to be on medication all the time, every day, so those all kind of affect my work…” (US-05)* • *“I found it hard to focus at work.” (US-12)*
**Social life impacts**	
**Isolation, reduced social interaction, reduced social life** • Includes inability to stay up late due to fatigue	• *“I think that’s always been the toughest part of it, is the emotional aspect of feeling so isolated.” (US-15)* • *“When I went out with friends and everyone was drinking alcohol and I was not allowed to. And I felt as an outsider.” (DE-05)* • *“It has affected my social life. For example, at the gatherings with my fellow students. Things can be especially difficult if there is food or drinking involved. It depends on the situation, but I generally try to avoid attending…” (CN-02)* • *“I can’t…stay up late. I have to stick to a routine to keep the disease under control.” (CN-06)* • *“I lost many ‘friends.’” (IT-02)*
**Social commitments/family relationships**	• *“When I have something planned, then suddenly I can’t do it anymore.” (DE-02)* • *“And they [relatives] may tell their children not to play with me…And tell the children not to get close to me…” I: How did you feel when you heard that? “I wanted to leave. I’d better to be alone.” (CN-09)* • *“He [father] just decided to label me as someone who is become a little bit of an outsider from his perspective, right, not a full member of the family, not full son. So these things running through my mind all the time.” (US-02)* • *“If you have like family or relatives or friends, and if you were trying to get close to their children, you feel like you don’t want to make the parents feel bad that you might some way give the virus to their kid, so that kind of puts us in a stigma that we shouldn’t be getting too close to other people’s children.” (US-05)*
**Meeting new people/difficulty maintaining friendships**	• *“First, I withdrew a bit, that was of course a restriction, and then regarding food and eating. As to partner choice, at some point you like to consider having a new partner and I blocked completely at first. For the first three or four years there was nothing. You don’t want to transmit anything to the new partner, do you? So, I let it all develop slowly and when I then explained this, there was not really a lot of understanding.” (DE-04)* • *“Well, there’s always that rejection from certain people sometimes. I have talked to someone and I kind of lost a few friendships because of this, because I thought I was just being open. I guess the misinformation, how you get it, sometimes people don’t understand and they think that by just shaking your hand or just by maybe sharing or drinking from the same glass of water they’ll get it. So there are people that have just walked away from my life because of that.” (US-13)*
**Difficulties with intimacy and sexual relationships/dating**	• *“In a partnership it is difficult to explain this to the other person. You don’t say that in the beginning. If the woman can’t handle it, she may never want to see me again. It’s not always easy.” (DE-06)* • *“I have less sex.” (CN-06)* • *“Yes. My relationships do suffer because of it [hepatitis B].” (CN-07)* • *“Socially it’s affected me in a way that I’m not dating. Before I was open to dating and I was putting myself out there and I was on a dating site. I’m not doing that because I still don’t feel that I am as comfortable as I want to be.” (US-11)*

*CHB* Chronic hepatitis B, *CN* China, *DE* Germany, *I* Interviewer, *IT* Italy, *JP* Japan, *US* United States

Reported lifestyle limitations of living with CHB included reduced ability or desire to exercise (*“I used to do exercise, but now I can’t do it anymore at that level. I get tired immediately”*), dietary limitations (*“The illness has an impact on the liver and…I have changed my diet”*; *“My diet has to be healthy and light”*; *“No spicy food, seafood, snacks”*), and avoidance of alcohol intake (*“I have to avoid alcohol. I don’t mean to get drunk, but a glass of wine would be nice”*; Table 2 and Additional File 1: Supplementary Table 1). Participants also reported increased medical appointments and need for time off, unwillingness to share cutlery, inability to stay up late or donate blood, and reduced productivity at work due to fatigue. Where participants had not disclosed their CHB status to their colleagues, less of an impact on work life was reported.

Social impacts of living with CHB were isolation, strain on relationships, and problems with intimacy. These were either self-imposed (*“Others can stay up late, but…for my physical considerations…I try to go to bed early”*) or external (*“they [relatives] may tell their children not to play with me…and tell the children not to get close to me”*; *“I have lost someone who could have become a serious relationship. She read my physical exam report and her attitude changed”*).

### Impact of CHB-related social stigma

Some participants initially described different understandings of the term “stigma” (Table 3 and Additional File 1: Supplementary Table 2). However, once the interviewer provided a pre-agreed definition “to stigmatize someone is to treat them negatively because they are perceived to be different,” the term was recognized by all participants, who were then able to describe experiences of social stigma. Impacts of social stigma were judgment/prejudice; negative impacts on relationships with partners, family, or friends; lack of awareness and understanding from others; avoidance or exclusion by others; others not wanting to share food, drinks, or utensils; negative experiences at work or at a medical facility; and being denied career opportunities (Table 3 and Additional File 1: Supplementary Table 2).

**Table 3:** Selected comments reflecting participants’ understanding and experiences of CHB-related stigma

Concept	Participants’ comments
**Understanding of stigma**	
**Participants’ definitions of “stigma”**	• *“…a preconceived myth or judgment that people identify with it.” (US-11)* • *“…it’s being put in a certain category that’s not looked at in a positive light.” (US-14)* • *“Prejudices. You get pigeonholed.” (DE-03)* • *“Stigma is something where a person is labeled, judged by others and where the person is also somewhat excluded.” (DE-08)* • *“It means to ‘label’ something.” (IT-07)* • *“…you feel uncomfortable and ashamed to see people because someone is judging and commenting on you.” (CN-12)* • *“[People] may feel that others are isolating them in various ways because the disease is a contagious disease.” (CN-01)*
**Impacts of stigma**	
**Experienced judgment/prejudice**	• *“People who I don’t know so well. People I only greet on the street. And suddenly they keep their distance. Or warn others.” (DE-10)* • *“I felt I was being judged, as if catching hepatitis was my fault because I did something, I felt as if I was someone’s ‘plague spreader.’” (IT-03)* • *“When someone thinks about sexually transmitted diseases, they always see you as if you were the dirtiest, weirdest person in the world…” (IT-04)* • *“I have felt badly because other people judge me without knowing the reason why I have contracted hepatitis B.” (IT-02)* • *“The doctor said, ‘You are a hepatitis B carrier! You could cause hospital infections!’” (JP-02)*
**Experienced relationship problems (negative experiences with partners or family and friends)**	• *“Well, there’s always that rejection from certain people sometimes. I have talked to someone and I kind of lost a few friendships because of this, because I thought I was just being open.” (US-13)* • *“I had a date. And there was a point when I had to tell him and it was over very quickly because he judged me and stigmatized me. He thought I was ill and contagious. He completely cut off contact with me.” (DE-09)* • *“People know to keep a distance away from you. They avoid you no matter what. I have fewer friends now.” (CN-06)*
**Experienced lack of awareness/understanding from others**	• *“People in [the US] generally don’t really know even the difference between the different types. And the most common one talked about here is hepatitis C, which is commonly associated with IV drug use, unsafe sex practices, other unsafe behaviors, which in turn cause a lot of people to judge other people based on those preconceived notions.” (US-12)* • *“It’s not contagious through talking, or food. But people, obviously they don’t quite understand.” (US-06)* • *“I had to explain what it is first. When I have a partner, I have to explain.” (DE-03)* • *“No matter what type of the hepatitis is, people are scared of it, they are scared of being infected. Many people actually don’t know exactly the transmission mode of hepatitis, so they’re so scared of it. They will keep away from you if they know you have it.” (CN-01)* • *“Many people have misunderstandings about hepatitis B, so they will definitely have certain precaution or discrimination.” (CN-04)*
**Avoidance by others**	• *“He went as far as not wanting to sit at the same dining table with me…I noticed some weird behavior on my dad’s part.” (US-02)* • *“People who were good friends before are avoiding me now. They would never tell me that they are avoiding me but they don’t have time for me anymore. We don’t meet anymore.” (DE-10)* • *“I noticed that everyone held back a bit, and me too, of course and so we were not so close any longer.” (DE-04)*
**Negative experiences at work**	• *“…everyone [in the work team] held back a bit, and me too, of course and so we were not so close any longer.” (DE-04)* • *“During the physical exam, I found out there was another coworker who had the same disease. When I found out that we had the same hepatitis B condition, I started to see him differently. When I read his report, I could only imagine what other people would have thought when they read mine. I turned in my resignation after that and left the job. So, I think job hunting has been a very challenging thing.” (CN-02)*
**Exclusion**	• *“Prior to disclosing [my hepatitis B status], the person I was talking with was very excited to have me and was ready to accept me into their [college martial arts] program. But then once I revealed my status, I got a follow-up email several days later saying that there was just not going to be a spot.” (US-06)* • *“I invited friends for a BBQ but they didn’t have time but later I heard that they did a BBQ themselves but without me [I felt like] an outsider. Hurt. And then I don’t want to go there the next time because I feel unwanted.” (DE-10)* • *“People shut the door on you only because you had hepatitis B.” (CN-05)*
**Others not wanting to share food/drink or utensils, self-restriction around sharing meals**	• *“But I do feel, I remember one time that I went to a park and I meet somebody, it’s a girl, and we chat awhile…and she’s a little bit hungry and I offer her an apple. But she was really happy, and when…there was a phone call, in that conversation I must’ve mentioned my hep B with my family. And she overheard it and she stopped eating that apple.” (US-06)* • *“Some of my relatives, middle-aged and elderly women, are generally mean-minded. When I went to their house for dinner, they will prepare a set of cutleries for me, and then said that they are prepared for me. After I used them up, they throw away those cutleries.” (CN-09)*
**Denied opportunities**	• *“I was trying to register classes in the college as a registered nurse major, and I tried really hard. And before you get enrolled, you need to provide them the proof of vaccination. And because I just noticed before the registration that I am a carrier, I tried to explain to them because I’m pretty healthy and I don’t have any symptoms…and they show me the instructions on where I can get treatment. And then I get treated, then I get to the vaccinations, but I can show them the proof of vaccination, and they won’t do that. So that’s really bothering, at one point, for me. It changed my career, actually. I really want to be a nurse, but I couldn’t because of this.” (US-06)* • *“I did pass multiple interviews and got to the last test. But I was failed because of the medical examination…Since then I only looking for jobs at those companies which don’t require the medical examination.” (CN-10)*
**Negative experiences at a clinic/hospital**	• *“Then the doctor might have seen my patient record and knew that I have hepatitis B. I accidentally touched some things, and I didn’t sit on the operating table as required by him. Then there was a nurse. She said, ‘You have hepatitis B, you don’t sit around, don’t transmit it to others.’ At that time, I felt very ashamed. She was so brutally candid and talked to me like that, I was shocked.” (CN-11)*
**Reduced social activities**	• *“…my usual social activities have been greatly reduced. Now I usually try to avoid going out to eat with others. I don’t want to join such activities.” (CN-09)*

*CHB* Chronic hepatitis B, *CN* China, *DE* Germany, *I* Interviewer, *IT* Italy, *JP* Japan, *US* United States

### Impact of CHB-related self-stigma

Participants were asked to describe their understanding and experience of self-stigma (Table 4) and its impact. Most participants provided a definition of self-stigma without help, except in Japan, where most participants had not heard of the term or were unable to offer a definition. This may have been due to the term not having a direct translation to Japanese (“stigma” was translated as “psychological distress”). Once the interviewer provided a pre-agreed definition (*“self-stigma is any time you think negatively about yourself based on what other people might think about you because of your hepatitis B”*), all participants, including those in Japan, understood the term.

**Table 4:** Selected comments reflecting participants’ understanding and experience of CHB-related self-stigma

Concept	Participants’ comments
**Understanding of self-stigma**	
**Participants’ definitions of “self-stigma”**	• *“Self-stigma is like, a classification that one has put upon himself. It’s not necessarily dictated by society, it’s internal and it’s how you feel about yourself. And a lot of times it’s probably a false perception, self-stigma. I mean, you may think everyone thinks I have the cooties and actually that could be in your mind, no one is judging you.” (US-14)* • *“It’s the stigma that we place on ourselves. It’s the thoughts and the feelings and the emotions that we have related to something, a title or a feeling.” (US-09)* • *“If I have a negative association about myself.” (DE-06)* • *“Perhaps it’s what happened to myself when I stigmatized myself, meaning, I self-blamed, I [pause] self-punished, that’s what I mean.” (IT-05)* • *“I feel ashamed about myself and avoid getting close to others because of my disease.” (CN-10)* • *“It means you are embarrassed because of your illness. It feels that you are less than other people.” (CN-02)*
**Impact of self-stigma**	
**Secrecy/concealment of diagnosis from all but trusted family or friends**	• *“…the reason I say it’s made me slightly self-conscious because it’s something that I’m not very comfortable with sharing with others and right there lets me know that it’s something I’m not proud of because of that reluctance to want to tell others that I have it. And, so, right there lets me know there’s an eternal internal reluctance or stigma that I have also placed on myself and not just society has placed it on me.” (US-14)* • *“Yeah, I wouldn’t want to tell anybody because I feel like people will treat me different and treat me like I’m a walking virus that’s contagious, that will hurt them. I can’t handle that.“ (US-11)* • *“I only talk about it to very few friends and I often withdrew from them.” (DE-01)* • *“My colleagues don’t know, my neighbors don’t know. I was embarrassed.” (DE-09)* • *“In daily life, there is no need to tell about my disease to distant relatives or friends you don’t normally interact with. I don’t want to give my wife’s relatives any worries, so I feel that I don’t have to tell them. However, if I was hospitalized for a long period of time, I wonder if I would have to tell them about the disease. It makes me anxious by telling people about my disease.” (JP-07)*
**Devaluation, self-blame, inferiority, worthlessness, shame, guilt**	• *“I think of that myself as not as good as other people…” (US-05)* • *“I judge myself. Because of my free life. I blamed myself.” (DE-09)* • *“Yes, feeling inferior, ‘dirty.’” (DE-04)* • *“I thought I was different than others.” (DE-05)* • *“I feel inferior to other people. I have ruined my life for not being careful. I blamed myself for it.” (IT-02)* • *“I feel I’m not good enough compared to other people…” (CN-02)* • *“I feel like a burden to my family. I’m rubbish, just like dust.” (CN-011)*
**Not taking opportunities**	• *“I’ve avoided some professions knowing that I wouldn’t want to deal with anyone knowing, such as like the medical field…And I think I remember in my early twenties reading that one story, it could have been just one in a thousand, but it definitely discouraged me from pursuing nurse practitioning, which is what I wanted to do for quite a while…” (US-12)* • *“I deliberately avoided these companies that require a medical examination.” (CN-10)*
**Withdrawal, social isolation, marginalization, alienation**	• *“I was depressed and withdrew into myself, and hardly went outside, only to work, stayed at home, only saw my very closest friends, one or two people or my mother. But otherwise I kept very much to myself.” (DE-04)* • *“At the beginning I couldn’t even imagine to kiss someone or get closer to someone. I withdrew and I was afraid. I didn’t want to infect other people.” (DE-09)* • *“I try to avoid social situations even if somebody ask me to hang out at night. And if I don’t want to let others know my hepatitis B, it’s just like, I just haven’t been completely honest with others, I’m hiding something from them.” (CN-10)* • *“When I have a choice of hanging out with my fellow students, I’d much rather be alone…” (CN-02)* • *“People who aren’t familiar with the disease like to avoid me.” (CN-07)*

*CHB* Chronic hepatitis B, *CN* China, *DE* Germany, *I* Interviewer, *IT* Italy, *JP* Japan, *US* United States

Concepts related to self-stigma mostly related to secrecy and CHB-status concealment, and concerns about other people’s perceptions. Responses included *“I wouldn’t want to tell anybody because I feel like people will treat me different [because they will think I’m] contagious”* and *“I thought of telling my friends but didn’t do it…I was afraid people would keep a distance from me”* (Table 4 and Additional File 1: Supplementary Table 3). One participant summarized their self-held belief as *“eternal internal reluctance or stigma”* they were constantly living with because of CHB. Feelings of low self-esteem, devaluation, and worthlessness were often expressed (*“I felt like I was different. I was ashamed. Especially at the doctor, because I always have to say that I have this illness”*; *“I feel inferior to other people”*; *“I feel like a burden to my family. I’m rubbish, just like dust”*). Shame and guilt were also reported by some participants (*“I have ruined my life for not being careful. I blamed myself for it”*), although others who were infected through blood transfusions or inadvertent contact did not feel self-blame (*“It’s not my fault…that I have this kidney disease or this hepatitis B”*). Participants described how self-stigma had resulted in withdrawal, social isolation, and avoidance of dating and social situations (*“At the beginning I couldn’t even imagine to kiss someone or get closer to someone. I didn’t want to infect other people”*; *“I don’t make too many friends because I am afraid of discrimination”*; *“I have to regulate myself from eating communally with non-family members”*).

Concepts regarding CHB-related self-stigma were more often reported by participants in China, Germany, Italy, and the United States, and rarely reported by those in Japan. This may have been not only due to language/conceptual differences but because many Japanese participants had not disclosed their HBV status to others (*“I don’t feel much about [self-stigma] because I don’t talk about the disease to others”*). Moreover, some participants in the United States and Germany whose CHB status had no impact on their work life added it was because they had not disclosed their CHB status (*“I haven’t had to submit blood samples or lab results. In [the US], employers…cannot do that for legal reason[s]. So…I haven’t been discriminated [against regarding] employment application[s]”*; *“I didn’t tell anyone about my illness, and I was always able to do my work properly”*).

Self-stigma–related feelings of fear, shame, embarrassment, secrecy, inferiority, and isolation seemed to result from social stigmatization. One reason for stigmatization is lack of awareness (or misunderstanding) of how HBV is transmitted or acquired (*“People [in the US] generally don’t really know the difference between [hepatitis] types. The most common one talked about here is hepatitis C, which is commonly associated with IV drug use, unsafe sex practices, other unsafe behaviors, which in turn cause a lot of people to judge other people [with CHB] based on those preconceived notions”*; *“I feel like people will treat me different…like I’m a walking virus that’s contagious, that will hurt them”*).

The participants reported not taking up career opportunities due to CHB-related social and self-stigma (*“I’ve avoided some professions [like the medical field], knowing that I wouldn’t want to deal with anyone knowing. I remember hearing a long time ago that there were medical schools that would not accept students with communicable diseases, especially hepatitis B”*; *“I can’t work in the industries involving food, such as supermarkets and childcare. I need to check liver function for this kind of work”*).

When asked of the impact of reducing self-stigma, most participants stated they would feel less worried about their future, less isolated, and that they could return to a “normal self” and “normal” lives, with reduced fear of rejection or judgment.

### Understanding of terms related to CHB severity and prognosis

Complete cure of CHB (i.e. eradication of viral covalently closed circular DNA) is not a realistic goal with current treatment options [33]; “functional cure,” defined as durable loss of hepatitis B surface antigen with or without seroconversion is considered the optimal treatment endpoint and is associated with significantly improved patient outcomes [27].

Participants were asked whether they were aware of and understood the terms “viral load” and “functional cure.” Up to two-thirds of participants could provide a definition of “viral load”; while none to half of participants in each country could provide an accurate definition of “functional cure.” No link was identified in any of the countries between participants’ education level or time since diagnosis and their understanding and awareness of either term.

In China, non-scientific terms “Little 3” and “Big 3” are typically used to characterize stages of CHB infection [34, 35]. “Little 3” refers to hepatitis B e antigen (HBeAg)–negative or HBeAg-positive status with low HBV DNA, whereas “Big 3” refers to HBeAg-positive status with high HBV DNA and higher risk of liver fibrosis. All participants from China were aware of these terms and able to discuss differences between, and implications related to, the 2 terms. Most participants believed “Little 3” was a better prognosis than “Big 3,” and some felt their CHB-related self-stigma increased if diagnosed with “Big 3.”

### Treatment expectations in relation to self-stigma

When asked how they would feel about a potential medication that could cure or achieve functional cure for CHB, the majority of participants who had self-stigma viewed it positively and felt it would reduce self-stigma (*“My self-stigma would change because if I don’t have it and it [can’t] be accounted for, and it’s not transmissible, I can go back to who I was. I don’t need to have the fear of rejection”*; *“Of course. I would feel like a normal person instead of a patient. I would no longer have a stigma”*; *“I would be more positive”*; *“I think it would be a beautiful thing, knowing that those thoughts about myself are gone”*; Additional File 1: Supplementary Table 4).

## Discussion

To understand impacts of CHB-associated social and self-stigma around the world, participants with a range of ages, treatment experiences, and socioeconomic status were recruited from countries in Asia, Europe, and North America to participate in this study. Findings showed CHB had wide-ranging impacts on all aspects of participants’ lives: social stigma was prevalent, affecting people in all countries included in this study, as was self-stigma, which was typically expressed as guilt, shame, and inferiority. Experiences of self-stigma included concealment of CHB status, social isolation, negative experiences at work and missed career opportunities, and relationship problems with family, friends, and/or intimate partners.

This study supported that, in all countries included, CHB-related social stigma is associated with strongly negative feelings, which typically manifest as self-stigma. Strong impacts of social and self-stigma were reported from all countries. Low self-esteem was widely reported, and most participants in all countries were cautious about disclosing their CHB status. When disclosed, it was only to close family or trusted friends.

These fears were not unfounded, as shown by these participants’ experiences and other reports. A survey among nurses in Japan revealed unwillingness to accept HBV/hepatitis C virus–infected colleagues due to stigma regarding infection routes and potential for nurse-to-patient transmission [36]. In China, although laws reducing access of HBV-infected people to employment and educational opportunities were amended between 2008 and 2010, stigmatization still occurs in workplaces, education, health care settings, and daily life [15, 22, 34]. Some employers continue to perform liver function testing through “health checks” [16], which further institutionalizes discrimination. Even in the United States, despite Centers for Disease Control and Prevention guidelines and legal protections, HBV-infected health care students face discriminatory policies, such as not being able to graduate medical school or restricted clinical training [5], with one respondent to a global Hepatitis B Foundation survey told to leave the US military due to HBV [14]. Participants in China reported the most concepts for each topic overall (with these topics very similar to those reported previously in a large sample [13]), which highlights persistent HBV-related stigma in China, especially in rural areas and in those with lower socioeconomic status [4, 22]. This is likely related to decades of sanctioned discrimination and culturally ingrained misperceptions about hepatitis B. By contrast, this study and previous studies showed people with CHB in Europe and North America are likely to experience overlapping, multiple levels of stigma based on social identities and discrimination (also called intersectional stigma), with HBV transmission frequently linked to high-risk sexual practices or sharing drug paraphernalia [4, 34].

Participants in all countries frequently characterized their experiences of social stigma as based on lack of understanding and fear of transmission. As shown in this and previous studies [13, 15, 20], social (and therefore self-) stigma leads to concealing HBV status, which can result in reduced medical follow-up or treatment adherence [6, 12, 22, 23] for fear of people finding out their CHB status. Increased, accurate public health messaging and education about HBV transmission would likely reduce social stigma [37] and therefore self-stigma [12]. Findings from this study and other studies also suggest a functional cure for CHB would be likely to reduce self-stigma and thereby substantially improve HRQoL [10, 38]. These findings will be used to develop a patient-reported outcome instrument to evaluate, among other resources, the impact on self-stigma and HRQoL of a treatment that achieves functional cure of HBV.

This study had several limitations. Specifically, identifying cultural effects on stigma-related experiences was not the objective of the study, and as participant numbers in each country were limited, country-specific conclusions cannot reliably be drawn. Nevertheless, common and country-specific themes emerged. In some countries, terminology used in this study had no direct or commonly understood translation in the relevant language. To address this, interviewers explained terminology to participants if they did not understand it before asking them to describe their experiences. Study strengths included offering telephone or web-assisted interviews to remove the anticipated barrier to participation in a face-to-face interview. The open-ended nature of questions ensured participants would volunteer descriptions of their experiences, so concepts and responses reported here emerged largely unprompted. Another strength was inclusion of participants from 5 countries from 3 geographic regions. Although the sample size for each country was relatively small, the total number of participants (for a qualitative study) of different ages, sexes, and socioeconomic statuses was relatively large and provided information from different cultural settings around the globe. Saturation achievement in the conceptual saturation analysis indicated that a sufficiently large sample was interviewed in this study.

In conclusion, CHB-related social and self-stigma are widely prevalent globally, often driven by lack of knowledge about HBV transmission routes and medical education regarding lifestyle limitations. The most common impacts of self-stigma included concealment of CHB status to avoid judgment and social isolation. Reducing self-stigma would help people with CHB feel more positive and less isolated and to return to their “normal” lives. Findings from this study support the concept that a treatment that could achieve functional cure would likely reduce social and self-stigma (particularly people’s fears about HBV transmission), which would improve HRQoL in people living with CHB. Considering both the impact of stigma on patients and the infectious nature of the disease could markedly change screening and treatment guidelines.

### Supplementary Information

**Additional file 1:** **Supplementary Table 1.** All quotes supporting concepts that emerged when participants were asked about their experience of living with CHB. **Supplementary Table 2.** All comments reflecting participants’ understanding and experiences of CHB-related stigma. **Supplementary Table 3.** All comments reflecting participants’ understanding and experience of CHB-related self-stigma. **Supplementary Table 4.** All participants’ comments reflecting the impact of a functional cure on self-stigma and the impact of reduction in self-stigma.

## Data Availability

The datasets supporting the conclusions of this article are included within the article and its additional files.

## Abbreviations

CHB: Chronic hepatitis B
HBeAg: Hepatitis B e antigen
HBV: Hepatitis B virus
HRQoL: Health-related quality of life
ISPOR: International Society for Pharmacoeconomics and Outcomes Research
