# Supplementary material for: Experience and impact of stigma in people with chronic hepatitis B: a qualitative study in Asia, Europe, and the United States
Source: BMC Public Health. 2024 Feb 26;24:611. doi: 10.1186/s12889-023-17263-6 (PMC10895774; doi:10.1186/s12889-023-17263-6)
Supplement: Supplementary file 1 — Additional file 1: Supplementary Table 1. All quotes supporting concepts that emerged when participants were asked about their experience of living with CHB. Supplementary Table 2. All comments reflecting participants’ understanding and experiences of CHB-related stigma. Supplementary Table 3. All comments reflecting participants’ understanding and experience of CHB-related self-stigma. Supplementary Table 4. All participants’ comments reflecting the impact of a functional cure on self-stigma and the impact of reduction in self-stigma. [file 12889_2023_17263_MOESM1_ESM.docx]

**Additional File 1**

**Supplementary Table 1. All quotes supporting concepts that emerged when participants were asked about their experience of living with CHB**

| Concept | Supporting quotes |
| --- | --- |
| **Emotional impacts** |  |
| **Anxiety/fear**  Reasons for feeling fearful included:   - Transmission to family and friends - Disease progression, including liver function and cirrhosis - Treatment efficacy - Impact on work - Impact on relationships/fear of rejection | - “I won’t touch people without thinking about my illness because I’m afraid that someone who knows about it might say, ‘Don’t do that.’ So I’m afraid of being an outsider. I don’t do things which are normal for other people. Hugging etc. or kissing I don’t dare to do that.” (DE-10) - “I avoid shaking hands or bodily contacts and I’m always careful to avoid someone else drinking from my glass.” (DE-01) - “I worry a lot about my liver. That it gets damaged…I’m quite young and I worry how many years I can live with it…I’m afraid of dying early.” (DE-09) - “I am afraid for my permanent job, how bad the illness is, whether I will get well again, whether the pills will help.” (DE-02) - “As to partner choice, at some point you like to consider having a new partner and I blocked completely at first. For the first three or four years there was nothing. You don’t want to transmit anything to the new partner, do you?” (DE-04) - “One is never free. It’s getting me down. I always feel like I’m in prison and I’m scared.” (DE-10) - I: So you are saying hepatitis B affects your contact with other people? “Yes, it makes me feel anxious, worried?” I: Why anxious? “That I can accidentally compromise the health of the people I’m with, for example through using the same cutlery, sneezing, things like that which perhaps are safe at the end of the day, as I was told it cannot be passed on that easily.” (IT-11) - “It’s like if…I’m not sure if it’s the right comparison…I had a time bomb in my pocket. I know I have it, but it’s unpredictable, so I do not know what could happen to me in the future or how it could affect my family, my children, my marriage.” (IT-11) - “Yes, definitely, especially in relation to my love and sexual life, if I can use this term, it would be much more relaxed. Beyond precautions, because I don’t want to get pregnant or similar, I almost avoid being touched because I’m scared to be contagious, so I find it difficult, very complicated. I have less fun, can I say it?” (IT-04) - “Yes, I understood. I probably wasn’t clear but it is ‘What will happen to me next?’ These two drugs I am taking all my life: Will they work? Are they not working? I go back every 6 months and the dose is increased. What does that mean? What will happen to me? How is it going to progress? What will happen next?” (IT-08) - “Because I know that hepatitis B is a disease that cannot be cured at present, and for people who have hepatitis B, if they fail to get proper treatment to control the disease, it will definitely have a significant impact on their life and work. So when I got the diagnosis initially, I was nervous.” (CN-01) - "I worry about cirrhosis. Hepatic fibrosis.” (CN-05) - “And I’m also worried about my condition getting worse. Because this disease is incurable currently, I just hope it isn’t getting worse.” (CN-10) - “The biggest concern is its transmission, as the disease is contagious.” (CN-09) - “First of all, the quality of life has greatly declined. It’s mainly because I feel afraid of infecting my family when I get home. Then I’m afraid that staying with my family for too long every day will increase the possibility of infection. In addition to my colleagues, I stay with my family for the longest time. I’m also afraid of saliva contact, and sometimes skin contact or blood contact, more or less contact, can make me afraid.” (CN-11) - “…Before I gave birth, I used to worry about my child. I don’t worry as much anymore.” I: What did you worry about? “Genetics and breast feeding.” (CN-07) - “Some companies require physical exam results before official hiring. Especially research type of jobs.” I: Have you experienced such a rejection so far? “No.” I: But it worries you? “Yes.” (CN-08) - “Also, while I take medication, because I have to take medications daily, it is inconvenient. I am worried people may see me taking medications at work.” (CN-02) - “ …some companies now do not require employees to take medical examination for hepatitis B, these companies may still require them to do so privately. If they are found to have hepatitis B, they may be fired…” I: And are you worried? “Of course.” (CN-10) - “Having hepatitis B…how should I say it…it makes job hunting very challenging, that is for sure.” (CN-02) - I: How did you feel at the time? “The name of the disease itself was special and unfamiliar to me. I didn’t know what kind of disease it was, whether it could be cured or not, or why I got hepatitis B. I have a wife and a child, so the thought that my treatment might be prolonged and I might miss work, quit my job, or worse, my life might be at stake has caused me tremendous anxiety. It was mentally damaging for a while because I had to figure out how I was going to live my life.” (JP-07) - “I’m concerned about the future. I go to the hospital regularly for follow-up.” (JP-05) - I: How does living with hepatitis B make you feel? “It’s scary.” I: How? “There is a risk of progression from cirrhosis to liver cancer.” (JP-11) - “Emotionally, it seems like I have a chronic disease because right now it’s not curable, so I just feel like I have a chronic disease. It might stay with me for my whole life. Yeah, that’s why I’m a little bit worried and kind of a little bit stigma like I have chronic disease.” (US-07) - “Socially, I do think about being very cautious not to possibly spread this to my kids or anything when I’m using utensils around the house or, you know, I have two boys—two younger kids, 12 and 13 so I try to worry about things like that.” (US-14) - “So, whenever I get into an accident, or have a cut, any type of situation where I think that my blood could be out in the open, I get very, very anxious and have sometimes avoided activities where I think that might happen.” I: What sort of activities would those be? “Sport, mostly sport, any kind of contact sport. And then sometimes even things like going hiking or camping, anywhere where I think I might have a possibility of injuring myself.” (US-12) - “When I cut myself, if I cut myself when I’m around people I try to clean myself up. I’m very high with my hygiene just because I know it’s blood and if somebody else gets that—that’s the only way of transmitting.” (US-01) - “He did a blood test and it came positive. That’s how I was diagnosed. I was devastated because I was very scared, didn’t know what it was, didn’t know how I would handle it. So it’s been a quite long journey.” (US-13) - “But in the beginning it was really a challenge because when you’re living with a chronic issue, I think that’s the biggest thing, is that you need to learn how to adapt, and to overcome any emotional insecurity surrounding that, and knowing that this is not something to be ignored, and it’s not something that will impact my life to the point of no return as long as I acknowledge it and don’t try and stay in that state of denial. I think there was a bit of denial of the beginning. There was a lot of insecurity, a lot of anxiety.” (US-15) - “Being with somebody else, it makes you better. So I believe in partners, partnership. I’m afraid that with what I have right now that’s not going to happen. Because I want to be truthful to anyone later on in the future and say, well, this is my condition. It’s scary because, to be honest, if I’m the other guy I’ll probably just run away from it, you know what I’m saying?” (US-13) - “My approach and self-confidence when it comes to forming relationships, may that be romantic, or friendship, or business related…I don’t know if it’s subconscious at this point because I’ve done a lot of work over the years to try to get peace with my condition. But sometimes it does show up in weird ways with my behavior where I’ll sort of sabotage myself before getting to a point where I have to take a risk or something like that just for fear of rejection, mostly I think.” (US-12) - “So, you add having hepatitis B on top of that, that’s a whole other level of transparency that you need to work with when you’re meeting a potential mate. And that’s very scary, that’s very insecure because there’s always that possibility of rejection, and very much so, and there have been people that I have met.” (US-15) |
| **Shame/embarrassment about disclosing condition**   - Includes shame related to inability to donate blood | - “At the beginning I felt ashamed of telling people because I don’t really know when I got infected and how. And then I keep wondering about it. And people are asking.” (DE-09) - “It affects me to a certain extent, as I told you before. I tell those who I trust. It is not a secret but, some people have prejudices, so I don’t tell them. Those who I tell, I don’t have any problem with them, also because I can’t hide it forever. I feel a bit embarrassed.” (IT-09) - “I would hide it at the beginning because of the fear of being negatively judged, being pushed away from the circle of people.” (IT-03) - I: …What other things are you capable of doing, but because of hepatitis B, it is difficult for you to try or do? “Blood donation.” I: You used to donate blood regularly, right? “Yes. Once every two years.” I: But now you dare not donate blood? “Yes.” (CN-09) - “For example, sometimes we see blood donation vehicles in the neighborhood and my friend asked when was the last time I donated blood? I did not know what to say.” (CN-02) - “It was a bit stressful for me that I have to tell my disease at the hospital or dentist. I felt ‘Why do I have to do this?’” I: What kind of stress is that? “It’s rather like a little embarrassing than feeling stressful.” (JP-01) - “It was embarrassing to have to take six weeks off and go into the HR manager of my job and show him the note. I said, ‘Can you keep it personal?’ I didn’t want anybody to know. I said, ‘I have to take a family leave,’ so I didn’t want anybody to know because then they look at you because people don’t know and they say, ‘Ooh, she’s contagious,’ so it was like fear, but now it’s much better.” (US-03) |
| - - - - 1. Low self-esteem | - “My self-esteem I think is a little bit down. And there are days where I just don’t want to see anyone or I don’t want to get out of the house, I just want to be alone. And I just go and do my own things.” (US-13) - “I think that’s always been the toughest part of it, is the emotional aspect of feeling so isolated and in feeling. Like I said, it’s gotten easier over the years…And I think that’s the biggest thing, is just getting to that point realizing ‘Hey, now, it’s time to continue to participate in my own life.’” (US-15) |
| - - - - 1. Need for secrecy/concealment | - “I also don’t tell everyone that I have the illness.” (DE-05) - “I never talk about my symptoms with my friends. So when I’m tired I blame other things for it.” I: So they don’t know about your illness? “No, definitely not.” (DE-11) - “There were moments when I didn’t want to tell people about my illness. That was an inner conflict.” I: And you wondered whether or not you should tell the person? “Yes.” I: Why? “Because I knew about the risks. But the risks applied to all people not just the people I trusted and told about the illness. I feel comfortable when I tell those people but other people I don’t tell about my illness. And there could be a situation that could potentially put the other at risk and that was a problem for me. Because I didn’t want to tell the other person but there could have been a dangerous situation.” (DE-08) - “Only my parents, my brother, and my girlfriend. Otherwise I didn’t tell anyone.” (DE-12) - “I told a good friend about it and he told everyone. And that was horrible. People were talking to me on the street and I denied it. I almost hit someone.” (DE-06) - “Well, no one knows about it, only my office colleagues know about it. Only my family knows, but no one else. My partner doesn’t know. And that is it. It has always been something I didn’t want to share or talk about. You are very careful in certain situation but you don’t tell it. You can’t tell your friends, unless it is a real and close friend. You don’t talk about this topic, never.” (IT-01) - “My friends have been told, not by me. I told one of my friends who then told them. They see me as someone to stay away from. So, I had several problems, as in, I lost many ‘friends.’” (IT-02) - “I didn’t tell everyone, I tried to hide it. I always managed to find different excuses not to do ‘tiring’ activities. Eventually, your friends start to leave you behind.” (IT-07) - “I would hide it at the beginning because of the fear of being negatively judged, being pushed away from the circle of people.” (IT-03) - “Some people have rich life experience, they see some of my symptoms, in fact, they are very conscious, people will ask me when they see my symptoms. Then they inferred that I had hepatitis B, but I didn’t admit it directly.” (CN-11) - “The medication doesn’t really affect the way I live. Not all of my coworkers know about my disease. When I take my medications at work, I have to peel the labels off…course not. I take my medication at the office only when there is nobody there. Otherwise I go to the bathroom, take the pills then come back out.” (CN-02) - “In terms of social life, because basically I won’t share or discuss my disease with others, it’s a private thing. If you don’t tell, nobody knows that you have hepatitis B.” (CN-01) - “Because most people in the society still have a biased view of this disease, I am worried that if they know that, it will affect our future relationship, so I can only be careful myself.” (CN-12) - I: Is there a reason, or can you tell me any reasons why you haven’t told those close to you that you have hepatitis B? “Oh, for my close family? Yes, they know, but not to just my colleagues and friends. I don’t want to scare them away…” (US-06) - “Well, you know, it’s something that I don’t—I mean, daily life I go with my life and people don’t know. There’s only a few people that I shared this information with.” (US-13) - “…And the reason I say it’s made me slightly self-conscious because it’s something that I’m not very comfortable with sharing with others and right there lets me know that it’s something I’m not proud of because of that reluctance to want to tell others that I have it. And, so, right there lets me know there’s an eternal internal reluctance or stigma that I have also placed on myself and not just society has placed it on me.” (US-14) - “Like, I’ve had workplaces where no one has known my status and I have never—I have never worried about it in that regard. But anytime I’m at a place and I know someone that I work with knows my status, I do look back at that one incident and sort of—I’m worried a bit sometimes about possibly being fired if my boss, you know, had some inkling in a different way to like wasn’t approving or something. But as far as like generally getting work done, it doesn’t affect me.” (US-12) - “Yes, in the past I guess that we worry about, not just me, many people worry about that we don’t want to disclose the condition. We might feel like we might get discriminated or might have been seen as different or been like feared by other people.” (US-05) |
| - - - - 1. Feeling overwhelmed, difficulty accepting diagnosis, sadness/depression, anger, betrayal, denial, not feeling human/normal | - “At the beginning it was very difficult for me to accept the diagnosis, because I could not understand how the infection came about. In the beginning it was very difficult for me psychologically.” (DE-08) - “I was lost when I got diagnosed. I didn’t know if I could be treated or if the disease can be controlled. I was overwhelmed.” (CN-03) - “I felt irritated and lost. I kept searching online for medications, I hoped so much there was a remedy that could cure it, turn it to negative, or just hide it, but there was not. You cannot hide this illness unless it is completely cured.” (CN-05) - I: Does hepatitis B cause you any trouble in daily life? “Big trouble! And this is so upsetting!” I: You mean fatigue or anything else? “Exhaustion is one of major troubles. But this illness also affects my appearance.” (CN-05) - “In fact, I was very sad and was uncomfortable at first, because I never thought I would get this disease. If I want to score my mood, it probably would be negative.” (CN-11) - “I took a long time off from work to focus on treatments. My bosses didn’t say anything but my coworkers speculated that I was seriously ill. They sensed I was seriously ill because I had to be off for two months. I was a little depressed.” (CN-07) - I: Has hepatitis B affected your mental health? “Sometimes I feel restless or stressed.” (CN-06) - I: Does it affect your mood? “It does. I try not to get angry…” (CN-08) - I: What are the changes on your mood? “Is it after I was infected?” I: Yes. “My temper will increase and I am easily explode.” (CN-03) - “Sometimes it can be such an emotional burden. I don’t consider myself a normal person anymore.” (CN-02) - “But in the beginning it was really a challenge because when you’re living with a chronic issue, I think that’s the biggest thing, is that you need to learn how to adapt, and to overcome any emotional insecurity surrounding that, and knowing that this is not something to be ignored, and it’s not something that will impact my life to the point of no return as long as I acknowledge it and don’t try and stay in that state of denial. I think there was a bit of denial of the beginning. There was a lot of insecurity, a lot of anxiety.” (US-15) - “…you become overwhelming because then they could say you could become chronic or God forbid the virus could spread so you know.” (US-03) - “…sometimes it can just be overwhelming to be on top of just knowing everything that’s going on with the hep B at the current moment.” (US-12) - “Like I mentioned, it makes me sad. I get depressed sometimes knowing that I have a disease. It’s inevitable. It makes me sad and depressed.” (US-01) - “Emotional, in the beginning very heavy on my shoulders. I was completely preoccupied. I felt like it was the end of my life.” (US-11) - “Yeah, sometimes I have. I go in depression mode.” (US-13) - “Ask me a month that it happened and I was, to be honest with you, a very upset, disturbed, and bothered person. I was irritable. I was angry.” (US-11) - “I was a little bit angry, I was concerned at finding out so late in life, and I was thinking, emotionally, would my husband understand, or will he not want to be with me because of it, so there were a lot of emotions that definitely came into play.” (US-09) - “It happened to me because of unprotected sex with someone that I really believed that I can live with a person forever. The person didn’t share that information with me. I felt like I was betrayed. Then that was hard for me to deal with that right now. There’s anger. Yeah.” (US-13) - “I felt betrayed. I felt abused by this guy that I had dated.” (US-11) - “I think there was a bit of denial of the beginning.” (US-15) - “It’s affected me emotionally by making me not feel human, making me not feel like I can do everything I want to do. It makes me feel like a lost soul. It makes me feel like just like I got this burden on me that I just can’t get rid of, and no matter what I do, I can’t shake it. So like I said before, it makes me feel kind of useless at time and whatnot.” (US-10) |
| - - - - 1. Lifestyle limitations |  |
| - - - - 1. Reduced/no exercise | - “Or in the past I went to the swimming pool and now I’m not doing that anymore.” (DE-10) - “I used to do exercise, but now I can’t do it anymore at that level. I get tired immediately. So, I had to slow down (go less times to) with the gym. So, now I go twice a week, when I go.” (IT-02) I: Has this been a problem to you? “Yes, because I am a very sporty and happy person. When I knew I was sick, I became a little depressed. I mean, I don’t see my life as it was before.” (IT-01) - “I used to do a lot of sport, but I don’t do it anymore.” (IT-07) - “Well, my lifestyle has changed, for sure, since I feel tired more frequently, I had to give up on a lot of sports, I used to do both tennis and swimming, I stopped swimming and I now play a bit of tennis, but not too much of it.” (IT-12) - “If you know a little about hepatitis B, you may know that patients with hepatitis B can’t be too tired, and can’t exercise vigorously, so, yes, it has.” (CN-12) - “Yes, it does have an impact. I tried very hard to find ways to keep myself as physically fit as healthy people, so I wouldn’t get tired so easily.” (CN-05) - “I can’t do any heavy house chores.” (CN-06) - “I used to run marathons. But when I run, there is a chance I could fall over and get some minor scratches. If you’re in good health, that’s not a problem. Even when I travel, I’m afraid of getting into the big common bath. It’s not that I’ve stopped traveling at all, but I’ve gone on fewer trips than before.” (JP-07) - “I was really pretty active. I mean, I go to the gym and I keep fit. And I did some research that in order to worsen the condition we’re not supposed to do very, you know, physically demanding even though it’s just for—it’s better for a short period of time, maybe 30 minutes, and not a very intensive one. So not being able to exercise as much as I could, I did gain some weight during the past year, about 10 years…I don’t want my body to be too exhausted and become weak to catch—I don’t want it to worsen my condition.” (US-06) - “I mean just like outside activities, you know, and trying to participate. You’re just trying to live life. I mean I could say like I don’t go swimming as much as I used to, I don’t jog as much as I used to. I mean it’s like any sort of extracurricular activity it takes a toll on, and it’s just like an extra weight that’s tearing you down that you can’t release.” (US-10) |
| - - - - 1. Dietary limitations and reduced/no alcohol intake | - “Eating has changed. The illness has an impact on the liver and I have to be careful in that respect. I have changed my diet and in various ways I no longer trust myself to do so.” I: I this change of diet a problem for you? “I have to do without certain things I used to like to eat.” (DE-01) - “First, I withdrew a bit, that was of course a restriction, and then regarding food and eating.” (DE-04) - “When I went out with friends and everyone was drinking alcohol and I was not allowed to. And I felt as an outsider.” (DE-05) - “I have to avoid alcohol. I don’t mean to get drunk, but a glass of wine would be nice at dinner. Fatty foods as well. I was told off by all the specialists.” (IT-07) - “Alcohol and food can trigger the virus. As it is dormant, in my case, I am very careful about food, fried food, fat meat, etc. Also, when I go out with friends, I drink very little. I don’t drink too much because of that.” (IT-06) - “My diet has to be healthy and light.” (CN-08) - “The impact on life is that I have to take medicines on time every day, have good rest and healthy diet, and also try not to be physically active.” (CN-01) - “No spicy food, seafood, snacks.” (CN-10) - “…The impact it has on living habits is that I need to pay more attention to my diet now.” (CN-04) - “It depends on the situation, but I generally try to avoid attending. I haven’t had a drink for a long time. If I have to entertain people, they have already been told that I’m allergic to alcohol. That’s my way out.” (CN-02) - “I can’t drink with my friends when we go out. Especially during special occasions such as weddings, parties, etc. People think I bring the party down because I don’t drink. These people don’t know about my disease.” (CN-07) - “I have decreased the amount of alcohol intake. Hepatitis B is the disease that affects the liver so drinking is not good for me. I don’t drink like crazy anymore.” (JP-06) - “I know that I shouldn’t overexert myself, rest and be too tired. I like to walk, so I try to improve my metabolism. Eat foods that are good for my body with a good nutritional balance in mind. I couldn’t drink coffee before but I’ve heard that drinking coffee after a meal is good for the liver, so I now try to drink it, but I can’t drink it on an empty stomach. Maybe it’s that coffee improves your metabolism and prevents fatty liver. I am very careful when I drink alcohol. I try not to drink too much alcohol and take supplements. Be careful not to get a hangover. I get drunk, but I’m conscious of my drinking. That’s about the only thing I’m aware of.” (JP-08) - “I never really drank much alcohol and now I won’t drink any because I always worry about the liver cirrhosis.” (US-03) - “But I don’t go out and drink anymore like I used to because of my liver and stuff like that. So every time I go out I just order a glass of water or something. I used to drink before and I used to love it. Now I can’t do that anymore because I’m always thinking about my liver. Because of the way that all of this is happening.” (US-13) |
| - - - - 1. Increased hospital visits, frequent treatment | - “When I had to go on dialysis, there were increased hygiene requirements for the nurses and patients. Last year I had a kidney transplant.” (DE-12) - “Yes, I always state it [hepatitis B status]; I had an appointment in the morning and once they saw on the form that I have hepatitis B, I was taken as the last patient.” I: I understand; so, at the most a bit of waiting and so on…? “Yes, one does have to wait a bit…” (DE-07) - “I am afraid that if I stop the medication, I will eventually develop cirrhosis of the liver. That’s why I take the medicine every day.” I: So this is a restriction. You have to think about the medication every day? “Yes.” (DE-06) - “It really affects my life. Every two months I need to go to the hospital to run all the tests to check the situation and how to address this issue according to the tests. Now I am taking the Zeffix and I am experiencing joint complications such as difficulties to take steps as side effects. When I get up, I feel pain everywhere. After blood circulation starts, I feel better. Still, it gives me muscular pain.” (IT-02) - “I take chronic therapies among which…can I name them? Deursil and Plaquenil, so…I will have to take them lifelong. Every 6 months, I repeat all the examinations: blood tests, ultrasound, etc.” (IT-08) - “The impact on life is that I have to take medicines on time every day.” (CN-01) - “The medications must be taken on an empty stomach or two hours after mealtime. Therefore, I cannot have a nightlife. I like to take my medications at nine o’clock and finish my meal at seven o’clock. It affects my social life.” (CN-07) - “My main trouble is that I take medicine every day. I’m worried that if I don’t take medicine, I will get worse.” (CN-12) - “I take a day off from work when going to the hospital. I could have taken the vacation days off and done something else if I didn’t have hepatitis B, but I have to give up on that idea. I am forced to pay a price.” I: How often do you go to the hospital? “I go to the hospital once a month on a weekday.” (JP-07) - “I have to go to the hospital four times a year, so it’s a lot of work. I have to keep taking these tests on and on. This is painful. There is also the pain of having an illness. I keep going to the hospital for tests and hearing the results, and it’s painful because I have to continue to do it. It’s a hassle and bothersome. But I have to receive an appropriate treatment and prescriptions to know what my current condition is like, so I guess it can’t be helped.” (JP-11) - “I have to go for blood tests frequently, but the virus hasn’t spread or chronic so I had a few shots of this [treatment] called interferon, but I just have to go for blood tests more periodically than the average person.” (US-03) |
| - - - - 1. Unable to share utensils | - “For example, if I have drunk from a glass or a bottle, I make sure that nobody else drinks from it.” (DE-01) - “I try to avoid giving my friends my glass for example.” (DE-06) - “Well, for example when I go to a lunch or a dinner, as I was told it can be passed on through biological fluids too, like saliva, so I have to be careful when I eat that nobody else uses my glass, things like that, maybe I’m exaggerated.” (IT-11) - “After knowing that I have hepatitis B, I usually use separate chopsticks when eating with them at home.” (CN-04) - “At the beginning, the house was divided into several areas, and then I would use independent tableware, and my dishes were also placed on a separate plate. I use a toilet by myself. My clothes or towels, including my cups, toothbrush, are all kept separately. In a separate area…” I: Okay, is that because you and your family have reached a consensus? Or is it because you do it on your own initiative? “I wanted to do it myself because there is a child at home. I am very worried that it will be transmitted to my child, and I had to do it.” (CN-11) - “For a while, I was scared when sharing a meal. We have always been cautious when we eat. For example, we use serving chopsticks when eating. To people who don’t know about my illness, they think we are so conscious about being healthy. To people who do know, they think that I’m being careless to share a meal while I’m sick. There is nothing I can do. Therefore, I’m very conscious. There is a lot of pressure.” (CN-06) - “Some of my relatives, middle-aged and elderly women, are generally mean-minded. When I went to their house for dinner, they will prepare a set of cutleries for me, and then said that they are prepared for me. After I used them up, they throw away those cutleries.” (CN-09) - “I try not to eat the hot pot communally and share their dishes with others. I usually eat on individual dishes and don’t share plates and utensils.” (JP-12) - “Socially, I do think about being very cautious not to possibly spread this to my kids or anything when I’m using utensils around the house or, you know, I have two boys—two younger kids, 12 and 13 so I try to worry about things like that.” (US-14) |
| - - - - 1. Impact on work - Taking time off work - Increased fatigue impacts performance   - - - 1. Some jobs no longer possible | - “At the beginning I had to get used to being more tired.” (DE-08) - “I had to take time off work because I was hospitalized. So, that period of time had an impact on my work life. But, again, I have not said the reason why I was off. Thankfully, there is a privacy law, that now digital certificated, the employer can’t see [pause] only the INPS (National Social Security Institute) can see your pathology, but not your employer. The employer can download the record, but he can’t see the diagnosis.” (IT-01) - “I get tired more easily. Fatigue is always there, so it has influenced my performance.” (IT-01) - “As far as work is concerned, since we are employed with fixed-term contracts, we have to undergo routine blood tests every year. In the past I had no problems if the test results were sent to my company e-mail address, I had no problems if somebody saw them. However, when the doctor informed, I suffered from this pathology I had to ask for the results to be sent to my personal e-mail address. When I did the tests last year…actually I’m due to repeat them this year and I’m quite anxious about it…I was sent the results to my personal email address, but the colleague I share my desk with told me she could see that everyone had received their results on their company email, but mine were missing. She found it strange as we did the tests together and was wondering why I did not receive the outcomes. I told her that maybe there was a problem, and I was going to call the lab to have the results sent. However, I can get away with it once, if the same happened next year and the year after that, I’m afraid people become suspicious.” (IT-11) - I: Has hepatitis B had an impact on your ability to do your job? “At first yes, because it made me tired. It brought on a little of depression.” (IT-05) - “I had to give up my job at the stables. Luckily, it was easy to find a job at that time. Now I would be probably begging for money in the streets or I could have found a job as a cleaner, that I couldn’t do because of my condition. I was lucky to know someone who helped me find a decent job.” (IT-07) - “I had to ask for a sick leave to my direct leader when I was in the hospital.” (CN-01) - I: …Are there any leave or absences due to hepatitis B or its treatment within the past two months? “Yes, I do. I was hospitalized for a week.” (CN-04) - “…right after my diagnosis. I did not go to work for half a year.” I: Because you had to go through treatments? “Treatments took some time. Also because my numbers were too high so I couldn’t go back to work.” (CN-06) - I: How did it affect your life and work? “I had to leave work for two months.” (CN-07) - “My biggest problem is not able to work for a long period of time without getting tired.” (CN-05) - “…but because I got hepatitis B, in fact, in many cases, I am unable to do as well as I would wish. So, my workload is reduced, my work efficiency is reduced, and my performance is not as active as before.” (CN-11) - “When I started working as an adult, I had to receive a medical checkup, so I always thought that I would probably not pass the paper exam because of hepatitis B. I avoided applying large corporations.” (JP-13) - “It’s affected me greatly. I got married right after graduating the university. I got pregnant, had a child and wanted to get a job but the fact that I have hepatitis B was always in my mind and couldn’t bring myself to find a job. They do a health checkup at a workplace, so it was difficult for me to motivate myself to get a job. I have been a housewife and had three children. I’ve never had a job.” (JP-15) - I: Does it affect you professionally or socially? “Of course, I am taking a half-day off from work when I go to the hospital, but since it’s only once every three months, so I think it’s necessary because it’s a way to keep track of my health.” (JP-05) - "I’ve been receiving treatment since my initial diagnosis, but I can’t help but have ebbs and flows in my symptoms. There are times when it doesn’t bother me so much. There are times when I get very tired and there are days when it’s hard to go to work. I work five days a week with regular hours and almost no overtime on flex time.” (JP-07) - “Yes, of course. I have to see the doctor, I have to visit, I had a liver biopsy before, I stay in the hospital. I have to see a doctor every other month or once every three months. I have to have lab tests all the time. I have to be on medication all the time, every day, so those all kind of affect my work efficient to my own physical problem.” (US-05) - “I found it hard to focus at work.” (US-12) - “I wouldn’t say it affected me to find a job but it definitely affected me to work, because like I said, the whole fatiguing factor. There have been times where I came up with an excuse that I had to bring a family member to a doctor’s appointment or something like that to get off work a little bit earlier because I was just feeling a little bit too fatigued and whatnot so it’s done that.” (US-10) - “I did take a leave of absence when this all happened because I was so checked out before asking these questions and I was not able to answer questions as I normally was because my mind was so preoccupied.” (US-11) - “No. Like I said, it was in the beginning more so that I had taken some time off to get through the interferon treatment.” (US-15) |
| - - - - 1. Social life impacts |  |
| - - - - 1. Isolation, reduced social interaction, reduced social life         2. Includes inability to stay up late due to fatigue | - “Yes, when I went out with friends and everyone was drinking alcohol and I was not allowed to. And I felt as an outsider.” (DE-05) - “Not necessarily from the girlfriend, but I think if strangers knew it, I would feel somewhat abandoned; after all it is infectious.” (DE-04) - “I don’t feel like doing anything then. I have no desire to receive guests, to go out, etc. I'm very passive.” (DE-11) - “I would rather stay at home instead of going out with those people I mentioned before, because I would be judged. It wouldn’t be an enjoyable moment; it would be quite unpleasant instead.” I: How do you feel when you don’t react to those people? “I feel misunderstood, because they should support me. If it had happened to one of my friends, I would have supported her. The opposite of what they have done.” (IT-03) - “It happened that I would shut myself off, I would avoid speaking or I would tell lies, ‘Everything is OK, I don’t have anything.’ I would hide it at the beginning because of the fear of being negatively judged, being pushed away from the circle of people.” (IT-03) - “I can’t overwork or stay up late.” (CN-07) - “The difference is that others can stay up late, but I have to try to participate in such activities as little as possible for my physical considerations. I try to go to bed early…” (CN-10) - “I can’t…stay up late. I have to stick to a routine to keep the disease under control.” (CN-06) - “Yes, it has affected my social life. For example, at the gatherings with my fellow students. Things can be especially difficult if there is food or drinking involved.” I: Do you prefer not attending? Or do you choose to attend but feel uncomfortable at the gathering? Which one? “It depends on the situation, but I generally try to avoid attending…” (CN-02) - “I wasn’t interested in doing anything. I didn’t want to go out.” I: No interest in social life? “Correct.” (CN-08) - “I cannot have a nightlife. I like to take my medications at nine o’clock and finish my meal at seven o’clock. It affects my social life.” (CN-07) - “I think that’s always been the toughest part of it, is the emotional aspect of feeling so isolated and in feeling. Like I said, it’s gotten easier over the years…And I think that’s the biggest thing, is just getting to that point realizing ‘Hey, now, it’s time to continue to participate in my own life.’” (US-15) - “I would tend to isolate because I was afraid of what they might think. I was afraid of what I thought of with myself because I had a lot of judgment towards myself, even though I was born with this. I had a lot of judgment with myself as a kid, and it seemed like—just like people’s lack of knowledge about it, I was afraid mostly of just how that first conversation would go or what they would think, even how I got it or anything like that. And as far as the communication goes, it’s hard to communicate to friends about something that they don’t really have a reference point to.” (US-12) - “Well, it affects me socially because in the same aspect of being active and all, you know, it’s like when will it take its toll on me next? When will I be at the mall with a few friends browsing some stores and then I just start feeling drowsy and fatigued and whatnot, and they’re not ready to go home yet and I would like to spend time with them. I would just like to be out of the house because I hate being inside the house, and I can’t do it all.” (US-10) |
| - - - - 1. Social commitments/family relationships | - “When I have something planned, then suddenly I can’t do it anymore.” (DE-02) - “I don't feel like doing anything then. I have no desire to receive guests, to go out, etc. I’m very passive.” I: So it has an impact on your social life? “Yes.” I: How much? Do you go out at all or only sometimes? “Rather sometimes. And I never talk about my symptoms with my friends. So when I’m tired I blame other things for it.” (DE-11) - “We used to go out and did a bit of partying, but that was limited for a while…” (DE-04) - “And they [relatives] may tell their children not to play with me…And tell the children not to get close to me…” I: How did you feel when you heard that? “I wanted to leave. I’d better to be alone.” (CN-09) - “And impacts on my family life. My wife went to the hospital immediately after knowing that I had hepatitis B and took an examination to see if she was also infected with hepatitis B. The result was negative, and then she went to the hospital to get the vaccine. The second injection was completed just two days ago…” I: You don’t have children yet? “Not yet. Because of hepatitis B, I may delay having children.” (CN-04) - “But some relatives don’t treat me nicely. Some of them avoid me and don’t let me visit them because I am contagious. We don’t visit each other anymore during New Year’s. This has affected me a lot.” (CN-02) - “I refrained voluntary from socializing such as going out for dinner that was not related to my job. I said, ‘I am sorry but I can’t go.’” (JP-12) - “He [father] just decided to label me as someone who is become a little bit of an outsider from his perspective, right, not a full member of the family, not full son. So these things running through my mind all the time.” (US-02) - “Yeah. And the other thing is if you have like family or relatives or friends, and if you were trying to get close to their children or things like that, you feel like you don’t want to make the parents feel bad that you might some way give the virus to their kid, so that kind of puts us in a stigma that we shouldn’t be getting too close to other people’s children as well.” (US-05) |
| - - - - 1. Meeting new people/difficulty maintaining friendships | - “First, I withdrew a bit, that was of course a restriction, and then regarding food and eating. As to partner choice, at some point you like to consider having a new partner and I blocked completely at first. For the first three or four years there was nothing. You don’t want to transmit anything to the new partner, do you? So, I let it all develop slowly and when I then explained this, there was not really a lot of understanding.” (DE-04) - “Every time in a particular situation, such as going to the dentist, going to get a blood test done or having a relationship with someone, yes, in those circumstances it would show itself. It still happens to this day; just because it regressed a little doesn’t mean anything. I always have this [pause] presence next to me that makes me reflect.” (IT-05) - “This has affected me a lot. The ones who are good to me, like a particular friend of mine, has always been nice to me. The ones who don’t treat me nicely see me as an infectious patient.” I: From what you just said, it seems that those relatives or the friends who aren’t so understanding, are treating you with some type of bias. “Yes. The bias is definitely there. Everyone can find out the definition for hepatitis B. They know they cannot be passed on through saliva. However, most people are still very scared about hepatitis B.” (CN-02) - “It has affected me. In the past, people consider it a contagious disease. People know to keep a distance away from you. They avoid you no matter what. Now that I have lived with it for so long, I have learned that hepatitis B could be transmitted through blood. That to me is not as contagious. Same as AIDS, which is also transmitted through contact with blood. If I was scared of people with AIDS, then I can understand why people are scared of people with hepatitis B like me.” I: I see. Therefore, the resistance is always there. “Yes. I have fewer friends now.” (CN-06) - “Well, there’s always that rejection from certain people sometimes. I have talked to someone and I kind of lost a few friendships because of this, because I thought I was just being open. I guess the misinformation, how you get it, sometimes people don’t understand and they think that by just shaking your hand or just by maybe sharing or drinking from the same glass of water they’ll get it. So there are people that I have noticed that they have just walked away from my life because of that.” (US-13) |
| - - - - 1. Difficulties with intimacy and sexual relationships/dating | - “As to partner choice, at some point you like to consider having a new partner and I blocked completely at first. For the first three or four years there was nothing. You don’t want to transmit anything to the new partner, do you? So, I let it all develop slowly and when I then explained this, there was not really a lot of understanding.” (DE-04) - “…with a partner, I should always indicate this [hepatitis B], and always use a condom as it is infectious unfortunately. This is what is uncomfortable, and at that time my girlfriend of course found out about this, too…” (DE-04) - “When I get closer to someone, sexual encounters, at first with condoms anyway, but at some point, then I have to tell my partner that I have this disease.” (DE-09) - “When choosing a partner, the question is always: Does it work the way I want it to? If you get to know each other better, the illness may be a hurdle, as you are not sure how the other person will react when you tell them about the illness.” I: You’ve been in situations like this? “Yes.” I: Did those situations occur when you met a person or when you were intimate with them? “It was more about intimacy. After getting closer to that person. Because then you need to tell the other person about the illness.” (DE-08) - “In a partnership it is difficult to explain this to the other person. You don’t say that in the beginning. If the woman can’t handle it, she may never want to see me again. It’s not always easy.” (DE-06) - “In a love relationship, it does influence me. In a social context with other people, it doesn’t. But I don’t talk about it, I have never mentioned that, if not my family.” (IT-01) - “If you are in a long relationship with someone, and you don’t use protection, in that case, the other person needs to be informed, so that she can vaccinate or similar. If not, intercourses have to be protected. As I am aware that the disease could be transmitted, it wouldn’t be fair on my partner. So, the disease has an impact in that sense, in romantic relationship.” (IT-06) - “In fact, it is generally acknowledged that there is no intimate relationship.” I: Now have you and your husband come up with a better plan or a consensus on this matter? Because I see that you are still quite young. “The only way is for the man to wear condoms. It’s the only way…Also, men and women would kiss at home, or drink the same cup of milk tea, same cup of drink, no big deal, including sharing towels and other daily necessities with lovers, now we can’t do that.” I: That is to say, you used to think it was romantic, but now you don’t dare to do it? “Yes.” (CN-011) - “I have less sex.” (CN-06) - “For daily work, it doesn’t make significant impact. But if it comes to privacy—” I: So when it comes to intimacy, it has a significant impact, right? “Yes, it does.” (CN-09) - I: …Has it affected your way of socializing or your dating? “Of course. It has affected my dating life. I have lost someone who could have become a serious relationship. She read my physical exam report and her attitude changed. She tried to make me feel better by telling me that the medication can cure the disease. But eventually she stopped contacting me.” (CN-02) - “Yes. My relationships do suffer because of it [hepatitis B].” (CN-07) - “Socially it’s affected me in a way that I’m not dating. Before I was open to dating and I was putting myself out there and I was on a dating site, on Match.com. I’m not doing that because I still don’t feel that I am as comfortable as I want to be.” (US-11) - “I’m single. So, that’s kind of an awkward area to approach when meeting people, when possibly meeting a partner or a mate, is how that conversation is going to come about. And that’s something that needs to be addressed. It needs to be addressed early on because I need to know if this is somebody that I’m getting involved with they can handle it, and also, you know, protection for them and, you know, just being aware.” (US-15) - “Yeah, if I’m with my partner in bed for the first time, then I just feel like oh my God, what happens if I tell, what would happen, would they say something negative about me or not.” (US-01) - “Well, you know, I stopped doing a lot of things. I’m having some issues right now because living with partner and stuff like that, it’s hard. The part, the intimate part, it’s struggling right now because of the relationship. I’m afraid that I can pass it onto that person. So I’m dealing with that right now.” (US-13) |

CHB, chronic hepatitis B; CN, China; DE, Germany; I, interviewer; IT, Italy; JP, Japan; US, United States.

**Supplementary Table 2.** **All comments reflecting participants’ understanding and experiences of CHB-related stigma**

| Concept | Participants’ comments |
| --- | --- |
| **Participants’ definitions of “stigma”** | - “Stigma is, I think, like a preconceived myth or judgment that people identify with it.” (US-11) - “For me stigma is like a title. Like your title, you’re a hepatitis person. To me that’s a stigma.” (US-01) - “Stigma is a…it’s being put in a certain category that’s not looked at in a positive light. It’s like you’re—yeah, you’re put in a category that’s not a popular category or one that’s looked at with, you know, abhorrence or indifference or, you know, it’s not—you don’t want to be in that particular category of how others look at you.” (US-14) - “I don’t know if it’s like this everywhere in the world. But in the United States, it seems like there is a certain stigma attached to the word ‘hepatitis.’ People in this country generally don’t really know even the difference between the different types. And the most common one talked about here is hepatitis C, which is commonly associated with IV drug use, unsafe sex practices, other unsafe behaviors which in turn cause a lot of people to judge other people based on those preconceived notions…” (US-12) - “Prejudices. You get pigeonholed.” (DE-03) - “How would I define this? Yeah, for example the attitude with AIDS and the drug addicts, or the fixers—that you are being pressed into some mold.” (DE-04) - “Stigma is something where a person is labeled, judged by others and where the person is also somewhat excluded.” (DE-08) - *“It means to ‘label’ something. I mean, it means to get close to someone and create an ‘avatar.’ I think it is not easy to explain.” (IT-07)* - *“It may mean that I’m a little afraid that other people will discriminate against me because they know I have hepatitis B. This is my understanding.” (CN-04)* - “I think it may mean that you feel uncomfortable and ashamed to see people because someone is judging and commenting on you.” (CN-12) - *“…to put the bad word on the patient but it sure feels not so good.” (CN-03)* - *“I think stigma means that the patients may feel that others are isolating them in various ways because the disease is a contagious disease.” (CN-01)* - “Some people look at patients with hepatitis B with a prejudice eye. That’s the hard part. When people do so, you feel that you shouldn’t make your disease public and cause people anxiety.” (JP-08) |
| **Experienced judgment/ prejudice** | - “He went as far as not wanting to sit at the same dining table with me…I noticed some weird behavior on my dad’s part. So I think even to this day, as recent as last month I went back to see my parents. I can still see that there’s a little bit of a distance, that he will just kind of sit as far away from me as possible when we’re having meal.” (US-02) - “You hear hepatitis, people associated HIV and AIDS. Words and associations and judgments that people make, it was not something that I ever would have anticipated and it was hurtful. That is why I had to take a leave of absence from work because mentally of course I was stable but I lost my desire to eat, I was upset, I isolated myself, I didn’t want to talk to anybody. I felt betrayed. I felt abused by this guy that I had dated.” (US-11) - “Yes, as said I got the impression from some that it was my own mistake, that I should have been more cautious; I argued with some, particular with men, and said frankly if you were to visit a brothel here or the girls on the streets, it could happen to you, too or even something worse, in that way. You can try to protect yourself but it is a direct judgment.” (DE-04) - “People who I don’t know so well. People I only greet on the street. And suddenly they keep their distance. Or warn others.” (DE-10) - “People were surprised of course and they asked where I got it from. And I was a bit embarrassed to tell them that I lived a relatively open life…” I: Were there people who judged you? “Yes.” I: From your circle of friends? “Yes.” I: How did you feel? “Not so good.” (DE-09) - “I felt I was being judged, as if catching hepatitis was my fault because I did something, I felt as if I was someone’s ‘plague spreader.’” (IT-03) - “No, the only thing that I want to say is that, in general, when someone thinks about sexually transmitted diseases, they always see you as if you were the dirtiest, weirdest person in the world. Unfortunately, these things happen.” (IT-04) - “I have felt badly because other people judge me without knowing the reason why I have contracted hepatitis B. I heard people saying that I got it because I used to do drugs, so I got it because I used a syringe. This made me feel really bad because I didn’t expect it from people who know that I don’t do drugs. Also, the fact that they think I contracted it in that way made me lose the trust I had in those people.” (IT-02) - I: Since you were diagnosed, have you experienced any unfair treatments? “Of course, some people would say things to me. I try to avoid them if I can.” (CN-06) - “Although they don’t avoid you on the surface, they will wash their hands as long as it’s something you touched, and they don’t want to touch it. They deliberately picked at me, which made me feel very uncomfortable.” (CN-11) - “It causes discrimination. Because I live with my parents, my tableware is separate from theirs. If I accidentally hurt myself while cooking, I must stop bleeding immediately, so as not to spread the virus to others. And I usually have to be very careful about hygiene. All my daily necessities, such as towels, have to be placed separately.” (CN-09) - “I am a hepatitis B carrier, aren’t I? I was worried how hepatitis A would affect me on the top of hepatitis B. The doctor said, ‘You are a hepatitis B carrier! You could cause hospital infections!’ He found out that I am a hepatitis B carrier when he checked my blood and told me that I should have told him about my disease beforehand. He was shocked and caused a scene.” (JP-02) |
| **Experienced relationship problems (negative experiences with partners or family and friends)** | - “My approach and self-confidence when it comes to forming relationships, may that be romantic, or friendship, or business related…I don’t know if it’s subconscious at this point because I’ve done a lot of work over the years to try to get peace with my condition. But sometimes it does show up in weird ways with my behavior where I’ll sort of sabotage myself before getting to a point where I have to take a risk or something like that just for fear of rejection.” (US-12) - “…for hep B somehow, it’s like this big demon that, you know, that just really create a lot of complications with my relationship with my dad.” (US-02) - “Well, there’s always that rejection from certain people sometimes. I have talked to someone and I kind of lost a few friendships because of this, because I thought I was just being open.” (US-13) - “Alright, so I guess the most direct one is from my partner, the person that I dated before, that was a clear direct mention. For example when I told her that I have hep B and I told her that I’m on medication, I was hoping that I can get some sympathy that I am on treatment, but the reaction was different, it was totally the opposite.” (US-05) - “Yes. I had a date. And there was a point when I had to tell him and it was over very quickly because he judged me and stigmatized me. He thought I was ill and contagious. He completely cut off contact with me.” (DE-09) - “I would tell my partner before we are getting closer and most don’t want to hear any details. And then it’s the end of that relationship. But maybe it’s my problem because I don’t want to get closer to that man and I think it’s all too complicated.” (DE-10) - I: Have you ever been treated differently? “Not really. Maybe while having sex.” (DE-01) - “If the woman can’t handle it, she may never want to see me again. It’s not always easy.” (DE-06) - “After I got hepatitis B, I have seen my family and close friends with two polar opposite reactions. Of course, my parents, my significant other, or children have always been nice to me. But some relatives don’t treat me nicely. Some of them avoid me and don’t let me visit them because I am contagious. We don’t visit each other anymore during New Year’s. This has affected me a lot.” (CN-02) - “My own family also discriminates against me, but it’s not that serious. The main discrimination comes from relatives and friends. I usually feel this kind of discrimination when we’re gathered together.” I: Okay. So they have a different attitude towards you, right? “Yes. They are worried about being infected by me.” (CN-09) - “In the past, people consider it a contagious disease. People know to keep a distance away from you. They avoid you no matter what. Now that I have lived with it for so long, I have learned that hepatitis B could be transmitted through blood. That to me is not as contagious…I have fewer friends now.” (CN-06) - “It can be depressing because of sudden changes. I was in love, but I broke up because of this disease. As a result, I was shattered, and my attitude towards marriage became very negative.” (CN-09) |
| **Experienced lack of awareness/ understanding from others** | - “People may think it’s a transmission disease, so they think, ‘Oh, you have something. It might transition to me,’ so when you’re eating food or like chopsticks, they will think it might transmit. They don’t share those things.” (US-07) - “I don’t want to have to share this information and then be further injured by their rejection or their inability to understand it, and respect me for who I am and that this is just a part of my life. But then to define me by that and be like, ‘Oh.’ And like I said, it’s all about education and understanding instead of ignorance and fear.” (US-15) - “It’s not contagious through talking, or food. But people, obviously they don’t quite understand.” (US-06) - “Because not all people have the knowledge. Some people they might think, when they hear that, even though it’s not, it is serious, but it’s not contagious, so you’re talking, you’re not thinking like that, but some people, they don’t know if somebody hears that you have infection, and they’re already scared, so I don’t want that to happen.” (US-08) - “But some people I know asked me if it was contagious. I’ve experienced that…I had to explain how this is transmitted.” (DE-02) - “Maybe a little when I said it. I had to explain what it is first. When I have a partner, I have to explain.” (DE-03) - “…Especially because of people who tell others as if they want me to carry a sign that I’m dangerous.” (DE-10) - “Because no matter what type of the hepatitis is, people are scared of it, they are scared of being infected. Many people actually don’t know exactly the transmission mode of hepatitis, so they’re so scared of it. They will keep away from you if they know you have it.” (CN-01) - “Many people have misunderstandings about hepatitis B, so they will definitely have certain precaution or discrimination.” (CN-04) - “Like I just mentioned some older colleagues who have rich life experience, and then they know you have this disease. Although I didn’t admit it, if they need to touch the paper or other things I’ve touched, they will wash their hands after touching them…They also wash their hands after using shared things, even though they don’t say it, or in public places, things I touched, such as teacups or kettles. They will also…basically they will want to wash it clean.” (CN-11) - “In fact, I am not contagious. People who aren’t familiar with the disease like to avoid me.” (CN-07) - “It’s almost as if I’m just spontaneously thinking about it. Some people may be biased. If you look up information about diseases on the Internet, it will come up immediately. I just think that they have a prejudice without looking it up, so I don’t deal with them. I try to push away their biased view towards me by saying, “If you don’t want to talk to me, you don’t have to.” (JP-07) |
| **Avoidance by others** | - “I’m sure I have. And like I said, there are some people that might have found out in a roundabout way through others, that might be friends of friends. And I don’t know how they found out. And yeah—I mean, it’s—they might not want to touch what I’m touching. They might not want to be around too close in contact.” (US-15) - “Yes. I can think of a few instances in college where I was—I signed up for—I believe it was a martial arts club, but I would not have been in contact with other people. It was just the individual. And I felt for some reason I needed to disclose my status. And prior to disclosing, the person I was talking with was very excited to have me and was ready to accept me into their program. But then once I revealed my status, I got a follow-up email several days later saying that there was just not going to be a spot.” (US-06) - “He went as far as not wanting to sit at the same dining table with me…I noticed some weird behavior on my dad’s part. So I think even to this day, as recent as last month I went back to see my parents. I can still see that there’s a little bit of a distance, that he will just kind of sit as far away from me as possible when we’re having meal.” (US-02) - I: Who knows about it? “My parents, my siblings, and unfortunately people who were good friends before but they are avoiding me now…They would never tell me that they are avoiding me but they don’t have time for me anymore. We don’t meet anymore.” (DE-10) - “I noticed that everyone held back a bit, and me too, of course and so we were not so close any longer.” (DE-04) - “Not to my colleagues. I told my family and my friend who spread the rumor and so my other friends became aware of it as well.” I: As you have told me before, you felt judged then. “Yes of course.” I: How did you feel about it? “I didn’t explain them how I got it, so I don’t know what they thought about the way I contracted it. They have just pushed me away. That’s it.” (IT-02) - “I was embarrassed at the beginning, because people say, ‘You’ve got hepatitis, stay away from me.’ This has been the impact at the beginning, the fear of telling people that I had it, but then I told myself, ‘What on earth am I doing this for?’ It’s not written anywhere that I have to pass it on to you, we don’t have to sleep together, we don’t have to drink from the same glass, so I relaxed, but that was the impact at the beginning.” (IT-03) - “Between colleagues I wouldn’t know after they found out then what will be their reaction but more or less, mentally they will have the rejecting idea. Some of them will show in action, might try to keep the distance. They do not know that this is a way of getting infected therefore they are not willing to have any close contact with you right?” (CN-03) |
| **Negative experiences at work** | - I: Did it cause any restrictions in the work team? “Yes, that did happen. I noticed that everyone held back a bit, and me too, of course and so we were not so close any longer.” (DE-04) - “I have changed my work. Before that I worked closer to my home. Then I changed work. Now my colleagues know about it. They don’t like it but in the sales room I can work normally.” (DE-10) - “During the physical exam, I found out there was another coworker who had the same disease. The coworker had already been there for many years, but I was just a newbie. That coworker seemed to be doing well and like a normal person. When I found out that we had the same hepatitis B condition, I started to see him differently. When I read his report, I could only imagine what other people would have thought when they read mine. I turned in my resignation after that and left the job. So, I think job hunting has been a very challenging thing.” (CN-02) - “[At] work. They will discuss how I get this disease, how I have sex with my husband, I may not be able to have a second child, etc…Those people get together when they are bored. They all have their own small groups.” (CN-011) |
| **Exclusion** | - “Yes. I can think of a few instances in college where I was—I signed up for—I believe it was a martial arts club, but I would not have been in contact with other people. It was just the individual. And I felt for some reason I needed to disclose my status. And prior to disclosing, the person I was talking with was very excited to have me and was ready to accept me into their program. But then once I revealed my status, I got a follow-up email several days later saying that there was just not going to be a spot.” (US-06) - “I invited friends for a BBQ but they didn’t have time but later I heard that they did a BBQ themselves but without me.” I: How did you feel? “Like an outsider. Hurt. And then I don’t want to go there the next time because I feel unwanted.” (DE-10) - “Mentally, I think this illness is a stumbling block definitely want to be 100% cured. It is more like a lion in the path. People shut the door on you only because you had hepatitis B.” (CN-05) |
| **Others not wanting to share food/drink or utensils, self-restriction around sharing meals** | - “But I do feel, I remember one time that I went to a park and I meet somebody, it’s a girl, and we talk about and chat awhile about lunch time, and she’s a little bit hungry and I offer her an apple. But she was really happy, and when I was on the phone, there was a phone call calling, so in that conversation I must’ve mentioned my hep B with my family. And she overheard it and she stopped eating that apple.” (US-06) - “And it’s very sad that I have those kinds of negative feelings because I know what to expect, seeing my dad running away from me. He’ll grab his food before I have a chance to touch the food and put it on his plate, right, and without even looking at me.” (US-02) - “…with one of my friends that I’ve known over the years when he became aware of the fact that I did have hep B, he didn’t abandon me, you know, we still socialize, drink beers and everything, but I noticed that he’s very cautious as to not, you know, getting our beers mixed up if we’re drinking.” (US-14) - “My family always use serving chopsticks. And I have my own eating utensils.” (CN-10) - “For a while, I was scared when sharing a meal. We have always been cautious when we eat. For example, we use serving chopsticks when eating. To people who don’t know about my illness, they think we are so conscious about being healthy. To people who do know, they think that I’m being careless to share a meal while I’m sick. There is nothing I can do. Therefore, I’m very conscious. There is a lot of pressure.” (CN-06) - *“Some of my relatives, middle-aged and elderly women, are generally mean-minded. When I went to their house for dinner, they will prepare a set of cutleries for me, and then said that they are prepared for me. After I used them up, they throw away those cutleries.” (CN-09)* |
| **Denied opportunities** | - “I was trying to register classes in the college as a registered nurse major, and I tried really hard. And before you get enrolled, you need to provide them the proof of vaccination. And because I just noticed before the registration that I am a carrier, so I tried to explain to them because I’m pretty healthy and I don’t have any symptoms…and they show me the instructions on where I can get treatment. And then I get treated, then I get to the vaccinations, but I can show them the proof of vaccination, and they won’t do that. So that’s really bothering, at one point, for me. It changed my career, actually. I really want to be a nurse, but I couldn’t because of this.” (US-06) - “But my wife said that for certain jobs, hepatitis B patients won’t have an opportunity.” (CN-04) - “My work. I did pass multiple interviews and get to the last test. But I was failed because of the medical examination…I’ve just experienced once. Since then I only looking for jobs at those companies which don’t require the medical examination.” (CN-10) - “I heard that the government canceled the hepatitis B examination couple of years ago, but in some special areas they do still check it up, so not 100% canceled. And it is so upsetting that my former profession does not tolerate any hepatitis B patient.” I: So that is why you changed your job? “Exactly.” I: You were interested in the job you were doing back then? “It was definitely the career I liked to pursue. And it really drove me mad that the profession which suits me the best would not let me take part because of my hepatitis B, and for some works which would not mind my illness, I was simply not able to do.” (CN-05) - “It does limit me while job hunting. Some jobs require physical exams.” (CN-08) |
| **Negative experiences at a clinic/hospital** | - “Then the doctor might have seen my patient record and knew that I have hepatitis B. I accidentally touched some things, and I didn’t sit on the operating table as required by him. Then there was a nurse. She said, ‘You have hepatitis B, you don’t sit around, don’t transmit it to others’…At that time, I felt very ashamed. In fact, the hospital must be a dirty place…But as a medical staff, you know bacteria is everywhere, you can’t talk to the patient this way. She was so brutally candid and talked to me like that, I was shocked.” I: …Did you talk to her and try to make it clear? “I dare not say it. If you say it, their attitude toward you will be even worse. If your operation does not go well, then you will be in a bigger trouble.” (CN-11) |
| **Reduced social activities** | - “I don’t socialize anymore. I hardly go out.” (CN-06) - *“…my usual social activities have been greatly reduced. Now I usually try to avoid going out to eat with others. I don’t want to join such activities.” (CN-09)* |

CHB, chronic hepatitis B; CN, China; DE, Germany; I, interviewer; IT, Italy; JP, Japan; US, United States.**Supplementary Table 3. All comments reflecting participants’ understanding and experience of CHB-related self-stigma**

| Concept | Participants’ comments |
| --- | --- |
| **Participants’ definitions of “self-stigma”** | - “I think just feeling inadequate, and it’s a self-pity and that sometime, people have a sense of guilt at how they get it.” (US-02) - “Self-stigma is like, a classification that one has put upon himself. It’s not necessarily dictated by society, it’s internal and it’s how you feel about yourself. And a lot of times it’s probably a false perception, self-stigma. I mean, you may think everyone thinks I have the cooties and actually that could be in your mind, no one is judging you.” (US-14) - “[Self-stigma] means the way that we think of ourselves.” (US-08) - “It’s the stigma that we place on ourselves. It’s the thoughts and the feelings and the emotions that we have related to something, a title or a feeling.” (US-09) - “It’s like bad talking to yourself. Like you’re negatively thinking bad about yourself because of the disease you have.” (US-01) - “If I have a negative association about myself.” (DE-06) - “For me this means that I no longer accept myself as I am because of my illness. There is also self-flagellation. That means people who think they did something bad, punish themselves for it. They are very strict with themselves because they can’t forgive themselves. So for me this is also a form of self-stigma.” (DE-08) - “I judge myself. And I did especially at the beginning. Because of my free life. I blamed myself.” (DE-09) - “I haven’t heard the term yet but I can imagine that you no longer see yourself as a complete person or you see yourself at the edge…So a self-perception, how one sees oneself, which can also be wrong.” (DE-12) - “It is a negative feeling that I have.” (IT-02) - “Perhaps it’s what happened to myself when I stigmatized myself, meaning, I self-blamed, I [pause] self-punished, that’s what I mean.” (IT-05) - “Self-esteem, for example. I started to believe less in myself when I realized I couldn’t push myself. I felt inferior to the others.” (IT-07) - “I feel ashamed about myself and avoid getting close to others because of my disease.” (CN-10) - “It makes a person feel like they are less than other people. It also feels like they are being cast away.” (CN-08) - “It means you are embarrassed because of your illness. It feels that you are less than other people.” (CN-02) - “It means that because I have infected with this disease, people talked about and I just identify with this general idea.” (CN-03) - “Self-stigma. It may indicate that hepatitis B patients are unwilling to tell others that they have hepatitis B and want to hide it.” (CN-04) - “Self-stigma comes from one’s self instead of from other people.” (CN-08) |
| **Secrecy/ concealment of diagnosis from all but trusted family or friends** | - I: And is there a reason for that? That you don’t let many people know? “I probably out of subtle embarrassment. I don’t want to be judged because then the questioning comes up, how I got it and, you know, people wonder if it’s contagious and they don’t want—it’s probably more social thing, how I’d be perceived by others, others around me. So I try to keep it, you know, on a low—kind of low keyed.” (US-14) - I: And do your colleagues know about your hepatitis B? “No.” I: And why is that? “It’s just a privacy thing, you know, so I don’t have to tell them that I have hepatitis B.” (US-08) - I: So can you tell me about how any self-stigma–related thoughts have affected you telling others that you have hepatitis B? “It’s not much related, actually, because I don’t think people around me, they don’t know. You know, just friends and they don’t know I’m hep B.” (US-06) - I: Is there a reason, or can you tell me any reasons why you haven’t told those close to you that you have hepatitis B? “Oh, for my close family? Yes, they know, but not to just my colleagues and friends. I don’t want to scare them away.” (US-06) - “…And the reason I say it’s made me slightly self-conscious because it’s something that I’m not very comfortable with sharing with others and right there lets me know that it’s something I’m not proud of because of that reluctance to want to tell others that I have it. And, so, right there lets me know there’s an eternal internal reluctance or stigma that I have also placed on myself and not just society has placed it on me.” (US-14) - “Yeah, I wouldn’t want to tell anybody because I feel like people will treat me different and treat me like I’m a walking virus that’s contagious, that will hurt them. I can’t handle that.” (US-11) - “Yes, in the past I guess that we worry about, not just me, many people worry about that we don’t want to disclose the condition. We might feel like we might get discriminated or might have been seen as different or been like feared by other people.” (US-05) - “…I know that people get afraid to go near you…” (US-03) - “The other person would just think about, well, am I in danger when I’m close to you, and that makes me feel even worse.” (US-05) - I: Tell me about how self-stigma–related thoughts have affected you telling others that you have hepatitis B. “Yes. I only talk about it to very few friends and I often withdrew from them.” (DE-01) - I: Tell me about how self-stigma has affected you being around others, apart from looking for a partner. “No. But not everybody knows about it. Only my closest friends and family and they are OK with it. My colleagues don’t know, my neighbors don’t know.” I: Have you not told about your illness because of the self-stigmatization? “Yes. And I was embarrassed…” (DE-09) - I: Tell me about how self-stigma–related thoughts have affected you telling others that you have hepatitis B. “Yes, I only tell people I trust.” (DE-05) - “I also thought that maybe I would lose friends because they didn’t understand.” (DE-03) - “I told a good friend about it and he told everyone. And that was horrible. People were talking to me on the street and I denied it…” (DE-06) - I: What about friends and colleagues? “No, not at all. I don’t want to tell them, as they would think that I am contagious.” (IT-08) - I: Have you been judged or treated differently by other people? “I didn’t tell everyone, I tried to hide it. I always managed to find different excuses not to do ‘tiring’ activities. Eventually, your friends start to leave you behind.” (IT-07) - “I mean, I go out with people whom I have met recently. I am talking about my closest friends with whom I don’t go out anymore. If I make new friends, I don’t tell them I have hepatitis, because it would prevent me from going out with them.” (IT-02) - “Only my relatives know my disease. Others don’t know it. Not even my friends, colleagues, and schoolmates.” (CN-10) - “Yes. I don’t like to disclose it. When I just gave birth, my friends asked if I was sick. I told them just the usual reason. However, some people found out about my disease, such as our accountant who approved my hospital bills. My direct supervisors know as well. I don’t like to disclose it to everyone because of my self-stigma. I don’t want to tell everyone.” (CN-07) - “Yes. I thought of telling my friends but didn’t do it…I was afraid people would keep a distance from me. It’s also not necessary.” (CN-08) - “In terms of social life, because basically I won’t share or discuss my disease with others, it’s a private thing. If you don’t tell, nobody knows that you have hepatitis B.” (CN-01) - “I never tell anyone. I feel it unnecessary to tell other people. Why shall I tell them?” (CN-05) - “I don’t feel much about [self-stigma] because I don’t talk about the disease to others. Isn’t it possible that hepatitis B can be passed on through sexual intercourse? So one might speculate that that’s why some patients were infected by it.” I: Could someone be hurt because of a lack of understanding around them? “Yes.” (JP-05) - “In daily life, there is no need to tell about my disease to distant relatives or friends you don’t normally interact with. I don’t want to give my wife’s relatives any worries, so I feel that I don’t have to tell them. However, if I was hospitalized for a long period of time, I wonder if I would have to tell them about the disease.” I: Is it depressing to think about the day you have to talk about it? “I don’t want to be concerned about me. I just want to be left alone. I don’t have that concern for the people that already know about my disease, but it makes me anxious by telling people about my disease.” (JP-07) - “I don’t tell people about my disease, so I’m not particularly affected by those around me.” (JP-05) - I: Have you ever been treated differently because you have hepatitis B? “No, because I haven’t told about it to anyone.” (JP-15) |
| **Devaluation, self-blame, inferiority, worthlessness, shame, guilt** | - “I think of that myself as not as good as other people…” (US-05) - “Yeah I do because I’ve had—especially in the first few years, and I was thinking, ‘Why did this happen to me?’ I don’t even know why I’m getting this. So it is—so it’s like I just feel that I’m not a normal person.” (US-06) - “It’s affected me emotionally by making me not feel human, making me not feel like I can do everything I want to do. It makes me feel like a lost soul. It makes me feel like just like I got this burden on me that I just can’t get rid of, and no matter what I do, I can’t shake it. So like I said before, it makes me feel kind of useless at time and whatnot.” (US-10) - “I do feel dirty sometimes, like you have a disease, you’re a walking disease.” (US-01) - “I judge myself. And I did especially at the beginning. Because of my free life. I blamed myself.” (DE-09) - “Yes, feeling inferior, ‘dirty’ which is silly but it’s like that.” (DE-04) - “At the beginning I thought I wouldn’t be able to live normally, I need to take medication. I felt negatively about taking medication daily. I thought I was different than others.” (DE-05) - “That’s difficult. I don’t like to talk about my illness…I think that people don’t understand me or misunderstand me and think negatively about me.” (DE-02) - “I felt like I was different. I was ashamed. Especially at the doctor’s, because I always have to say that I have this illness. For example, at the dentist I always thought that the doctor would use high doses of disinfectant. I also thought that maybe I would lose friends because they didn’t understand.” (DE-03) - I: Any self-blame? “No.” I: Or maybe where you got it from? “I suspect it happened in hospital. I myself was in hospital when I was 10 years old and a little boy hit his head and bled and I helped him.” I: But you don’t feel bad that you helped him? “No.” (DE-03) - “When you have such an illness you think: Why me? Why not the others? And in my case with the blood transfusion I thought: How can this actually happen nowadays to get such an illness through a blood transfusion?” I: Do you also blame yourself, even if it has happened to you as part of your medical care? “No. It’s not my fault. It’s not my fault that I have this kidney disease or this hepatitis B.” (DE-12) - “Yes, I felt badly because of my condition. I feel inferior to other people.” I: Could you make me a practical example, for me to better understand? “I have ruined my life for not being careful. I blamed myself for it.” (IT-02) - “Yes, maybe I would feel unpleasant if I had to tell someone who [pause]. As long as I tell doctors, friends, relatives, it’s fine. I would feel unpleasant if I had to tell someone who doesn’t have to know. I would feel a bit inferior, a bit different.” (IT-09) - I: You were also afraid of telling them. Would your life be different if you did not have a bad temper or become suspicious because of hepatitis B? “Without it I would probably be a normal person like I was.” (CN-08) - “I’m used to it by now. I didn’t like it when I just got diagnosed. I felt inferior and wasn’t happy about it.” (CN-06) - “I feel I’m not good enough compared to other people…” (CN-02) - I: Have you had self-stigma for such a long time? In what ways? “I feel inferior to others.” (CN-09) - “I feel like a burden to my family. I’m rubbish, just like dust.” (CN-011) - I: I see. So do you think your hiding of your disease has caused you a certain psychological burden? “Yes. It makes me feel guilty.” (CN-10) - “Yes, in the beginning I was a little depressed. I felt bad about catching the disease and couldn’t change anything.” (CN-08) - “I also feel a little bit guilty, because this disease is not good for either myself or my family.” (CN-012) - I: Please rank the importance from one to five. “Feeling ashamed/guilty because I have hepatitis B.” I: Why is this one the most important to you? “I can’t tell people that I have this disease, I don’t want to, and I don’t want them to know.” I: Is it because of a lack of understanding? “Yes. They don’t understand this disease properly.” (JP-05) |
| **Not taking opportunities** | - “I guess I can answer in the fact that I’ve avoided some professions knowing that I wouldn’t want to deal with anyone knowing, such as like the medical field, I had sort of—and I know every school doesn’t do this, but I remember hearing a long time ago that there were medical schools that would not accept students with communicable diseases, especially hepatitis B. And I think I remember in my early twenties reading that one story, it could have been just one in a thousand, but it definitely discouraged me from pursuing nurse practitioning, which is what I wanted to do for quite a while…” (US-12) - I: Had you avoided work when you first got diagnosed with hepatitis B? “I had to pick and choose the industry when I job hunted.” (CN-08) - “I deliberately avoided these companies that require a medical examination.” (CN-10) - I: Do you personally think that this hepatitis B will impact your ability to work in the future or your ability to find other jobs in the future? “I can’t work in the industries involving food, such as supermarkets and child care jobs. I’m not young anymore, and I need to check liver function for this kind of work. So I can’t work in such industries.” (CN-09) |
| **Withdrawal, social isolation, marginalization, alienation** | - “I was depressed and withdrew into myself, and hardly went outside, only to work, stayed at home, only saw my very closest friends, one or two people or my mother. But otherwise I kept very much to myself.” (DE-04) - “Yes of course. At the beginning I couldn’t even imagine to kiss someone or get closer to someone. I withdrew and I was afraid. I didn’t want to infect other people.” (DE-09) - “I withdraw in certain situation and I lock my feelings. Then I’m not so active anymore and I don’t talk anymore…” (DE-01) - “I thought everybody is avoiding me.” (DE-06) - I: I would like to know if the fact that other people think you have hepatitis B influenced you, because they reacted negatively? “Yes of course, I feel influenced. I would rather stay at home instead of going out with those people I mentioned before.” (IT-02) - “I try to avoid social situations even if somebody ask me to hang out at night. And if I don’t want to let others know my hepatitis B, it’s just like, I just haven’t been completely honest with others, I’m hiding something from them.” (CN-10) - “I don’t make too many friends because I am afraid of discrimination. Because the more friends I have, the more things my friends know, the more likely they would find out that I have the disease.” (CN-10) - “When I have a choice of hanging out with my fellow students, I’d much rather be alone…” (CN-02) - “Yes. I thought of telling my friends but didn’t do it…I was afraid people would keep a distance from me. It’s also not necessary.” (CN-08) - “I’m afraid of my hepatitis B being found by others. Because if they know, they will be avoiding or rejecting me when I want to eat or hang out with them. So I try not to let others know.” (CN-10) - “Yes. In fact, I am not contagious. People who aren’t familiar with the disease like to avoid me.” (CN-07) - “I have to regulate myself from eating communally with non–family members. Not a single person I know has said anything about it, but there may have been some who felt uneasy about it.” (JP-09) |

CHB, chronic hepatitis B; CN, China; DE, Germany; I, interviewer; IT, Italy; JP, Japan; US, United States.

**Supplementary Table 4. All participants’ comments reflecting the impact of a functional cure on self-stigma and the impact of reduction in self-stigma**

| **Comments** |
| --- |
| - “My self-stigma would change because if I don’t have it and it’s basically not able to be accounted for, and it’s not transmittable, I can go back to who I was. I don’t need to have the fear of rejection, the fear of—I don’t even need to communicate it to anybody.” (US-11) - “Well, absolutely. I think anybody that’s ever walked around with a chronic illness like this, yeah, it definitely changes. It would probably get rid of those moments of that thinking of like, ‘Why me? How did this happen? Am I being punished?’ You know, because we all have those deep inherent thoughts when…you just don’t talk about it. So, it would completely eradicate that, in a sense. It would kind of be like having a new lease on life.” (US-15) - “Again, because of my condition or disease, I’m a little embarrassed so that embarrassment affects me psychologically and if there was a functional cure, I think I could live a much happier life and a much more content and quality of life is the number one thing in relation to—in relationship to your existence. So, it’s very important to get rid of the self-stigma phenomenon.” (US-14) - “I wouldn’t have some thoughts anymore. I would approach different things completely differently. My quality of life would be different.” I: And in relation to self-stigmatization. What would be different? “My thoughts would be more positive.” (DE-01) - “I wouldn’t have to think about it anymore, I would feel normal.” (DE-05) - I: How would you feel? “I wouldn’t have so much self-doubt anymore. I would be more self-confident.” (DE-12) - “My life would be better because when I wake up in the morning, I even struggle to walk because I feel pain everywhere. If there was a drug that could tackle the side effects, it would be better.” (IT-02) - “I think it would be a beautiful thing, knowing that those thoughts about myself are gone, thoughts about transmitting it to someone else.” (IT-03) - “They would change. Of course, they would change. I’d be freer. I’d feel more free and relieved from this burden I carry with me.” (IT-05) - “I think that I would feel a free and healthy person. I wouldn’t feel the burden of sharing the information with other people. I wouldn’t need to be careful with food and drinks. And my love life as well. So, there are many aspects involved. I would feel free of obligations, and free.” (IT-06) - “Of course. I would feel like a normal person instead of a patient. I would no longer have a stigma.” (CN-07) - “Maybe the feeling of self-stigma would decrease a little…Decreased self-stigma would make me think that I’m a normal person. I can hang out and live with others like a normal person. I would not feel guilty or have any psychological pressures.” (CN-10) - I: If a new treatment can attain functional cure, would it affect your self-stigma? “I would still be somewhat affected.” I: Would it reduce your self-stigma? “Reduce it by ten or twenty percent.” (CN-08) - “I can live like a normal person again. But because most people have never heard of such cures, even if I tell them that I have been cured, they may not believe it.” (CN-09) - “If it can cure hepatitis B, my stigma will definitely be reduced.” (CN-04) - “I want all viruses to be gone from my body. I want to be cured completely for once. That’s the most important aspect for me.” I: If that could ever come true, how would you feel? “I would be more positive.” (JP-13) |

CN, China; DE, Germany; I, interviewer; IT, Italy; JP, Japan; US, United States.
